# Blood Biomarkers of Alzheimer Disease and Rates of Global and Domain-Specific Cognitive Decline

**DOI:** 10.1001/jamanetworkopen.2026.28975

**Published:** 2026-08-13

**Authors:** Erika J. Laukka, Ingrid Ekström, Martina Valletta, Bengt Winblad, Claudia Fredolini, Sarah Andersson, Laura Fratiglioni, Davide Liborio Vetrano, Giulia Grande

**Affiliations:** 1 Aging Research Center, Department of Neurobiology, Care Sciences and Society, Karolinska Institutet and Stockholm University, Stockholm, Sweden; 2 Stockholm Gerontology Research Center, Stockholm, Sweden; 3 Division of Neurogeriatrics, Department of Neurobiology, Care Sciences and Society, Karolinska Institutet, Solna, Sweden; 4 Theme Inflammation and Aging, Karolinska University Hospital, Huddinge, Sweden; 5 Affinity Proteomics Stockholm, Science for Life Laboratory, Department of Protein Science, School of Engineering Sciences in Chemistry, Biotechnology and Health, Royal Institute of Technology, Solna, Sweden

## Abstract

**Importance:**

While evidence is accumulating on blood-based biomarkers of Alzheimer disease (AD) and future cognitive decline, community-based studies that include phosphorylated tau (p-tau) 217 are still scarce. Moreover, there is limited knowledge regarding associations with domain-specific cognitive decline and modifying effects of key individual characteristics.

**Objective:**

To investigate associations of blood biomarkers of AD with global and domain-specific cognitive decline.

**Design, Setting, and Participants:**

This prospective cohort study included participants from the Swedish National Study on Aging and Care–Kungsholmen recruited between March 21, 2001, and August 30, 2004. Participants were aged 60 years or older, provided blood samples, and were free of dementia at baseline. Follow-up assessments were completed on December 19, 2019. The statistical analysis was conducted between April 1 and September 30, 2025.

**Main Outcomes and Measures:**

Baseline biomarkers, including amyloid β 42/40 ratio, p-tau217, p-tau181, total tau, neurofilament light chain (NfL), and glial fibrillary acidic protein (GFAP), and cognitive performance (episodic memory, semantic memory, verbal fluency, perceptual speed) were assessed at baseline and across 15 years of follow-up. Linear mixed models were used to assess associations between biomarker quartiles and rates of change in specific cognitive domains and a global composite score. Interactions were explored for age, sex, and *APOE* ε4 status.

**Results:**

In the 2008 participants (mean [SD] age at baseline, 71.7 [10.1] years; 1215 female [60.5%]) included, associations with rate of change were observed across all cognitive domains for amyloid β 42/40 ratio (*P* for trend = .006), p-tau217, p-tau181, NfL, and GFAP (all *P* for trend < .001), but not total tau. Significant interactions were observed for *APOE*, for which higher levels of p-tau181 were associated with faster decline in ε4 carriers vs noncarriers (β = −0.037 [95% CI, −0.047 to −0.026] vs −0.020 [95% CI, −0.025 to −0.015]). In contrast, higher levels of NfL and GFAP were associated with faster cognitive decline only in non–ε4 carriers (β = −0.025 [95% CI, −0.031 to −0.020]) and −0.017 [95% CI, −0.022 to −0.012], respectively). Associations between NfL and GFAP and cognitive decline were exacerbated in females (β = −0.023 [95% CI, −0.029 to −0.018] and −0.021 [95% CI, −0.028 to −0.015], respectively).

**Conclusions and Relevance:**

This cohort study in a general older population found that AD biomarkers were associated with long-term rates of cognitive decline. The observed patterns varied across biomarkers and individual characteristics and suggest that sex and genetic risk of AD should be considered in the interpretation of these biomarkers.

## Key Points

**Question:**

What is the association of different blood-based biomarkers of Alzheimer disease (AD) with global and domain-specific cognitive decline in the general population?

**Findings:**

In this cohort study of 2008 older adult participants in the Swedish National Study on Aging and Care–Kungsholmen, AD-related biomarkers, especially phosphorylated tau 217, phosphorylated tau 181, neurofilament light chain, and glial fibrillary acidic protein, were associated with faster rates of cognitive decline over up to 15 years of follow-up. Associations differed depending on the biomarker measured and participant sex and *APOE* genotype.

**Meaning:**

Easily accessible blood-based biomarkers of AD may be linked to subsequent faster cognitive decline in the general older population, with patterns suggesting some heterogeneity across biomarkers and that individual characteristics may influence clinical interpretation.

## Teaser

This cohort study investigated the associations of blood biomarkers of Alzheimer disease with global and domain-specific cognitive decline among older adults participating in the Swedish National Study on Aging and Care–Kungsholmen.

## Introduction

Blood-based biomarkers of Alzheimer disease (AD) have emerged as easily accessible, noninvasive tools to identify individuals at higher risk of developing cognitive impairment and AD dementia.^1,2,3^ Especially phosphorylated tau (p-tau) 217 has shown great promise as a specific biomarker of AD, sensitive to early pathologic alterations in amyloid and tau deposition.^3,4^ In light of emerging disease-modifying therapies, more studies are needed on how different biomarkers could be used to monitor disease progression in the general population.

In AD development, brain alterations may appear years to decades before observable clinical symptoms,^5,6,7^ making extended follow-up essential to track cognitive change trajectories during this period. Moreover, specific cognitive abilities may be differentially sensitive to early dementia-related changes^8,9^ and reflect different types of dementia pathology.^10,11,12^ There is insufficient knowledge regarding the long-term associations between diverse blood-based biomarkers of AD and domain-specific cognitive decline.^13^ Many studies have limited the assessment to global cognition or memory,^14,15,16^ and studies targeting a wider range of domains have produced inconsistent results.^17,18,19,20^ Large sample sizes are needed to detect potential differential patterns, depending on the biomarker and cognitive domain assessed, and to investigate possible modifying effects of individual characteristics, such as age, sex, and genetic predisposition for AD. We therefore investigated associations between 6 blood-based biomarkers of AD and neurodegeneration, including amyloid β (Aβ) 42/Aβ40 ratio, p-tau217, p-tau181, total tau (t-tau), neurofilament light chain (NfL), and glial fibrillary acidic protein (GFAP), and rates of global and domain-specific cognitive decline over 15 years in a large population-based sample of individuals free of dementia at baseline.

## Methods

This cohort study collected data within the population-based Swedish National Study on Aging and Care–Kungsholmen (SNAC-K). The study was approved by the Regional Ethical Review Board (dnr 2023-02375-02) in Stockholm, Sweden. All participants, or proxies for those with cognitive impairment, provided written informed consent. The study adhered to the Strengthening the Reporting of Observational Studies in Epidemiology (STROBE) reporting guideline for cohort studies.

Residents of the Kungsholmen district in central Stockholm were randomly selected to take part in the study. Of 4590 alive and eligible persons, 3363 (73.3%) participated in the baseline assessment (March 21, 2001, through August 30, 2004). Participants belonged to predefined age strata and were called back every 6 years (age groups 60, 66, and 72 years) or 3 years (age groups ≥78 years). Follow-up was completed on December 19, 2019.

### Data Collection

Each data collection wave entailed a nurse interview, medical examination, and neuropsychological assessment. We included individuals free of dementia (*Diagnostic and Statistical Manual of Mental Disorders* [Fourth Edition] criteria) (eMethods 1 in Supplement 1), Parkinson disease, schizophrenia, and developmental disorder at baseline. Individuals who did not perform the neuropsychological assessment or had a Mini-Mental State Examination (MMSE) score of less than 24, indicative of cognitive impairment, were not included. Of those remaining, all included participants provided complete biomarker data (eFigure 1 in Supplement 1). Individuals who did not contribute biomarker data were more likely to be older, female, have a lower level of education, and have a lower MMSE score. Age was measured as years since birth and education as years of formal schooling. Inhabitants of the Kungsholmen district were generally White with a relatively high socioeconomic status; thus, these variables were not analyzed. Information on medical diagnoses was collected through the nurse interview, medical examination, medication lists, laboratory tests, and the National Patient Register (eMethods 2 in Supplement 1).^21^

### Blood Biomarkers and Genotyping

Nonfasting peripheral venous blood samples were collected at baseline. Upon centrifugation, serum aliquots were stored at the Karolinska Institutet Bio Bank at −80 °C in cryogenic storage vials until analysis. Protein quantification was performed at the Affinity Proteomics Stockholm Unit (SciLifeLab) using SR-X Biomarker Detection, version 1.2.0 software (Quanterix) (eMethods 3 in Supplement 1).^22^ Limit of detection values and coefficients of variation are reported in eTables 1 and 2 in Supplement 1. Data below the limit of detection were replaced with the value of 0 (5 for Aβ42, 12 for t-tau, 3 for p-tau217, and 12 for p-tau181).

A ratio was calculated for Aβ42/Aβ40, and the biomarkers values were *z* score transformed in the sample with data on all 6 markers (n = 2360). The continuous standardized variables were further divided into sample-specific quartiles, where quartile 4 (Q4) indicates the highest values. Higher quartiles imply higher levels of brain pathology for all markers except the Aβ42/Aβ40 ratio, for which the opposite is expected. *APOE* (rs429358) genotyping was performed using matrix-assisted laser desorption/ionization–time-of-flight analysis on the MassARRAY platform (Sequenom) at the Mutation Analysis Facility, Karolinska Institutet.

### Neuropsychological Assessment

A cognitive test battery was administered according to the same standardized procedure at each wave.^23^ All cognitive scores were standardized using the baseline sample mean and standard deviation as the standardization base. Domain scores constituted the mean of all standardized scores available for that domain.

Global cognition was calculated as the mean of all standardized scores when data were present for at least 2 domains. Episodic memory was assessed with recall and recognition of a 16-item word list.^24^ After presentation, participants were given 2 minutes for immediate free recall, and the number of correctly recalled words was recorded. Next, participants were given a self-paced yes-no recognition task consisting of the 16 target words randomly intermixed with 16 distractors. The recognition score was calculated as the number of hits (correct yes answers) minus the number of false alarms (incorrect yes answers). Semantic memory was assessed with the Synonyms Reasoning Blocks–Part 1 30-item multiple-choice Swedish vocabulary test.^25^ It was scored as the number of correctly identified synonyms within 7 minutes. For verbal fluency, participants were given 60 seconds to orally generate as many words as possible beginning with a certain letter (*F*, *A*) or belonging to a specific category (animals, professions). The perceptual speed tasks comprised 2 paper-and-pencil tests. In digit cancellation,^26^ the number of correctly crossed *4*s after 30 seconds was counted. For pattern comparison,^27^ the score was computed as the mean number of correct pattern classifications across 2 trials, both lasting 30 seconds.

### Statistical Analysis

Data were analyzed between April 1 and September 30, 2025. To account for expected nonlinear effects in the associations between the biomarkers and future rates of cognitive decline, our main analyses treated the biomarkers as categorical variables in the form of quartiles. The associations between the biomarkers and cognitive levels and rates of change were estimated using linear mixed models performed with StataSE, version 19.0 (StataCorp LLC). Years from baseline was used as the timescale. Fixed effects included individual biomarkers, time in study, and the interaction term for biomarker and time. Random intercepts and slopes were applied, permitting intercepts and slopes to vary across participants. We used unstructured variance-covariance matrices for all models. Missing data were handled through maximum likelihood estimation. In all models, we controlled for age and education, centered at the sample mean. We further controlled for sex and anemia, kidney disease, obesity, and cerebrovascular and cardiovascular disease, chronic conditions known to influence levels of blood biomarkers and cognition.^28^

As secondary analyses, biomarkers were modeled as continuous variables to ensure the consistency of the results and explore potential interactions. This approach retains statistical power, avoids arbitrary cutoffs, and enables testing whether associations vary across subgroups. To explore whether the associations for NfL and GFAP were present independent of level of AD-specific pathology, we applied a cutoff derived from a previous study from SNAC-K investigating the usefulness of blood-based biomarkers of AD for predicting 10-year dementia in the general older population.^22^ We used the cutoff for p-tau217 (0.134 pg/mL) to approximate which individuals had lower (n = 1310) vs higher (n = 698) levels of AD-specific pathology. To investigate whether estimates for rate of change were statistically different, we ran bootstrapping analyses using the packages nlme and boot in R, version 4.5.2 (R Foundation for Statistical Computing), comparing the β values for specific cognitive domains in the total sample. Finally, we checked for 3-way statistical interactions (variable by biomarker by time) and performed stratified analyses for *APOE* genotype, age, and sex. A threshold for significance of *P* < .05 was applied throughout the study.

## Results

A total of 2008 participants (mean [SD] age at baseline, 71.7 [10.1] years; 1215 female [60.5%] and 793 male [39.5%]) were included in the study. Baseline characteristics of the study sample are reported in Table 1. Participants were followed up for up to 15 years (mean [SD], 7.2 [5.4] years). They took part in neuropsychological assessments a mean (SD) of 2.6 (1.3) times and were tested on 1 (n = 527), 2 (n = 391), 3 (n = 626), 4 (n = 308), 5 (n = 122), or 6 (n = 34) occasions. Follow-up was shorter among participants in biomarker Q4 compared with Q1, independent of age, sex, and education (eg, p-tau217 mean [SD], 4.4 [4.7] vs 8.9 [5.0] years; NfL, 4.1 [4.3] vs 9.1 [4.9] years) (*P* < .001). Baseline characteristics for participants with and without follow-up data are provided in eTable 3 in Supplement 1. Correlations among all biomarkers and cognitive domains are shown in eTable 4 in Supplement 1.

**Table 1.** Baseline Characteristics of Study Participants for the Total Sample and by Age Group and Sex

Characteristic	Participants, No. (%)				
	Total (N = 2008)	Age group		Sex	
		<78 y (n = 1294)	≥78 y (n = 714)	Male (n = 793)	Female (n = 1215)
Age, mean (SD), y	71.7 (10.1)	65.2 (4.8)	83.5 (5.3)	70.4 (9.8)	72.6 (10.2)
Sex					
Female	1215 (60.5)	734 (56.7)	481 (67.4)	NA	NA
Male	793 (39.5)	560 (43.3)	233 (32.6)	NA	NA
Education, mean (SD), y	12.3 (4.2)	13.2 (4.2)	10.6 (3.8)	12.8 (4.4)	12.0 (4.1)
MMSE, mean (SD)	28.9 (1.3)	29.3 (1.0)	28.2 (1.6)	29.0 (1.2)	28.9 (1.4)
Anemia	195 (9.7)	53 (4.1)	142 (19.9)	96 (12.1)	99 (8.1)
Kidney disease	619 (30.8)	181 (14.0)	438 (61.3)	178 (22.4)	441 (36.3)
Obesity	275 (13.7)	206 (15.9)	69 (9.7)	113 (14.2)	162 (13.3)
Heart disease	409 (20.4)	145 (11.2)	264 (37.0)	192 (24.2)	217 (17.9)
Cerebrovascular disease	101 (5.0)	38 (2.9)	63 (8.8)	39 (4.9)	62 (5.1)
*APOE* ε4 carrier^a^	579 (29.6)	396 (31.5)	183 (26.3)	239 (31.3)	340 (28.5)
Laboratory assessment, median (IQR)					
Aβ42/Aβ40 ratio	0.06 (0.05-0.07)	0.06 (0.05-0.07)	0.05 (0.05-0.06)	0.06 (0.05-0.07)	0.06 (0.05-0.07)
p-Tau217, pg/mL	0.10 (0.05-0.17)	0.07 (0.04-0.12)	0.17 (0.10-0.28)	0.10 (0.06-0.18)	0.09 (0.05-0.16)
p-Tau181, pg/mL	1.15 (0.74-1.75)	0.93 (0.63-1.36)	1.73 (1.19-2.45)	1.23 (0.76-1.87)	1.10 (0.72-1.71)
t-Tau, pg/mL	0.84 (0.55-1.16)	0.75 (0.49-1.06)	0.97 (0.67-1.35)	0.80 (0.52-1.14)	0.86 (0.55-1.19)
NfL, pg/mL	17.34 (12.38-26.52)	13.89 (10.64-18.12)	29.43 (21.23-41.56)	16.89 (12.16-25.29)	17.58 (12.49-28.00)
GFAP, pg/mL	117.88 (77.75-178.39)	95.20 (65.48-131.91)	180.51 (130.58-269.00)	101.83 (68.32-150.52)	128.79 (87.49-200.72)
Cognitive assessment, mean (SD)^a^					
Word recall	7.1 (2.4)	7.7 (2.2)	5.9 (2.2)	6.8 (2.3)	7.3 (2.5)
Word recognition	11.6 (2.9)	11.9 (2.6)	11.1 (3.3)	11.4 (2.9)	11.8 (2.9)
Vocabulary	22.9 (5.0)	23.9 (4.4)	21.0 (5.5)	22.9 (5.0)	22.9 (5.1)
Letter *F*	15.3 (5.5)	16.0 (5.5)	14.0 (5.3)	15.2 (5.6)	15.3 (5.4)
Letter *A*	12.6 (5.1)	13.2 (5.0)	11.4 (5.1)	12.3 (5.0)	12.7 (5.1)
Animals category	21.3 (6.5)	23.3 (6.2)	17.8 (5.6)	21.4 (6.6)	21.3 (6.5)
Professions category	16.2 (5.5)	17.9 (5.1)	13.0 (4.7)	16.5 (5.7)	15.9 (5.3)
Digit cancellation	17.7 (4.3)	19.1 (3.9)	14.9 (3.8)	17.5 (4.3)	17.8 (4.3)
Pattern comparison	14.1 (4.0)	15.8 (3.1)	10.8 (3.4)	14.4 (4.0)	13.9 (4.0)

Abbreviations: Aβ, amyloid β; GFAP, glial fibrillary acidic protein; MMSE, Mini-Mental State Examination; NA, not applicable; NfL, neurofilament light chain; p-tau, phosphorylated tau; t-tau, total tau.

^a^ Missing data for *APOE* ε4 carrier (n = 54), word recall (n = 30), word recognition (n = 31), vocabulary (n = 19), letter *F* (n = 15), letter *A* (n = 21), animals (n = 7), professions (n = 9), digit cancellation (n = 65), and pattern comparison (n = 74).

### AD Blood Biomarkers and Rate of Cognitive Decline

The results from the linear mixed models based on quartiles are shown in the Figure (global cognition), eFigure 2 in Supplement 1 (specific cognitive domains), and Table 2. Lower Aβ42/Aβ40 ratio was associated with a faster decline in global cognition and across all examined cognitive domains, although effect sizes were relatively small. Higher quartiles (reflecting higher concentrations) of p-tau217, p-tau181, t-tau, NfL, and GFAP were each associated with faster global and domain-specific cognitive decline. Effect sizes were most pronounced for participants in Q4 of p-tau217, p-tau181, NfL, and GFAP (all *P* for trend < .001), exhibiting a 0.042-SD to 0.058-SD faster mean annual decline in global cognition compared with Q1 (Table 2). For each quartile increase, p-tau217, p-tau181, and NfL showed a dose-response association with faster decline (all *P* < .001, except for the comparison between Q1 and Q2: p-tau217, *P* = .02; p-tau181, *P* = .005). Sensitivity analyses further adjusted for *APOE* genotype and excluded participants with an MMSE score of less than 27, missing follow-up data, or incomplete data on the global cognitive score, showing very similar results as the main analysis (eTables 5-8 in Supplement 1).

**Figure.**
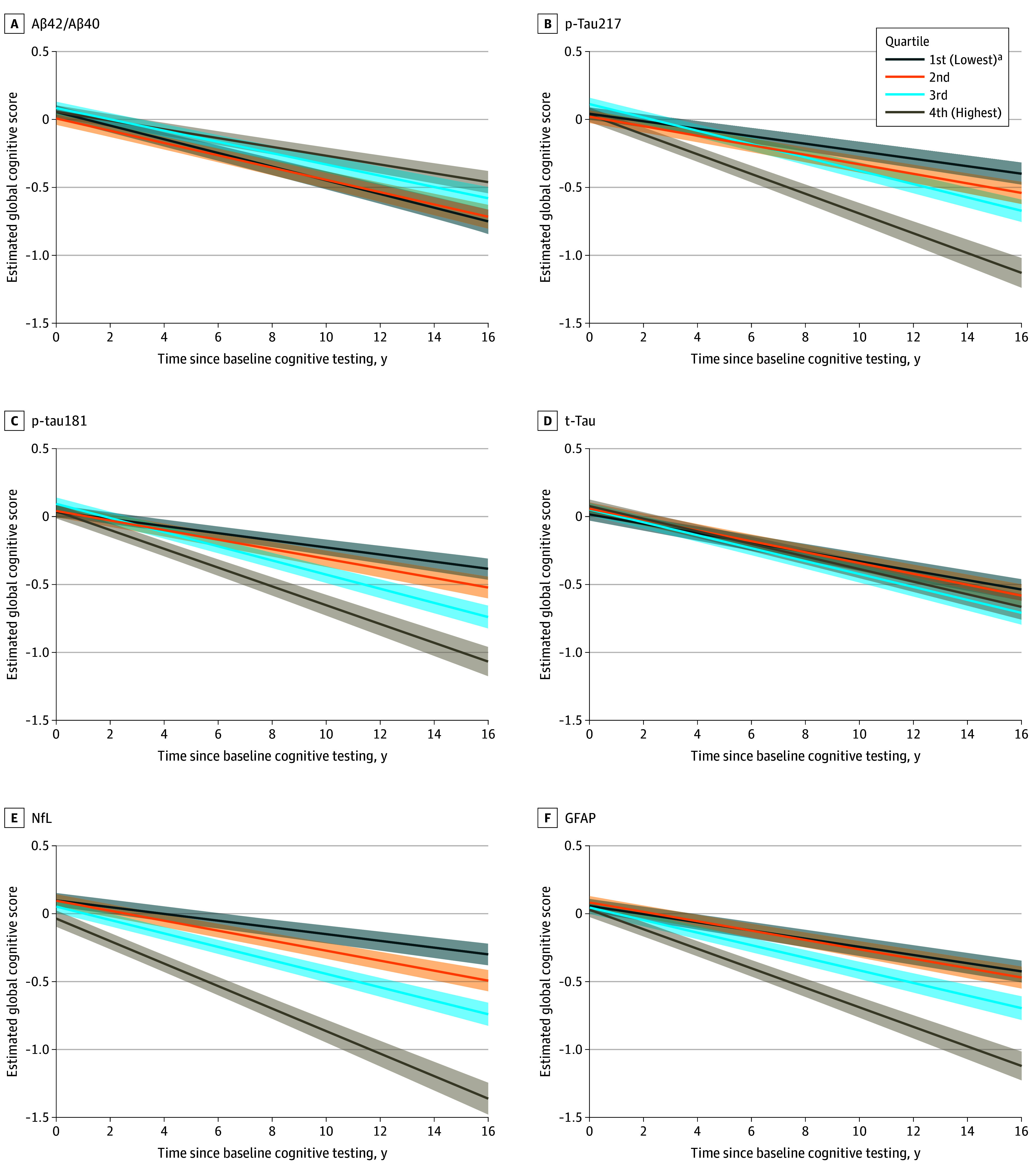
Line Graphs of the Estimated Trajectories of Global Cognitive Decline Across 15 Years for Quartiles of 6 Blood-Based Biomarkers of Alzheimer Disease All models were adjusted for age, sex, education, anemia, kidney disease, obesity, cerebrovascular disease, and cardiovascular disease. Shading indicates the 95% CI. GFAP indicates glial fibrillary acidic protein; NfL, neurofilament light chain; p-tau, phosphorylated tau; t-tau, total tau. ^a^For the amyloid β (Aβ) 42/Aβ40 ratio, the fourth quartile indicates the highest ratio and lowest amyloid burden. For all other biomarkers, the fourth quartile indicates the highest pathologic burden.

**Table 2.** Estimates From Linear Mixed Models by Quartiles of 6 Individual Biomarkers of Alzheimer Disease (N = 2008) for 4 Specific Cognitive Domains and Global Cognition

Biomarker	Cognitive domain, β (95% CI)^a^				Global cognition, β (95% CI)^a^
	Episodic memory	Semantic memory	Verbal fluency	Perceptual speed	
**Aβ42/Aβ40 ratio**					
Q3^b^	−0.012 (−0.024 to −0.000)^c^	−0.009 (−0.018 to −0.000)^c^	−0.005 (−0.013 to 0.003)	−0.007 (−0.015 to 0.002)	−0.009 (−0.016 to −0.002)^d^
Q2	−0.010 (−0.022 to 0.003)	−0.018 (−0.027 to −0.009)^e^	−0.012 (−0.021 to −0.004)^d^	−0.010 (−0.019 to −0.001)^c^	−0.013 (−0.020 to −0.006)^e^
Q1 (lowest ratio and highest burden)	−0.019 (−0.032 to −0.007)^d^	−0.016 (−0.026 to −0.006)^d^	−0.016 (−0.024 to −0.007)^d^	−0.015 (−0.024 to −0.006)^d^	−0.018 (−0.025 to −0.011)^e^
*P* value for trend	.006	<.001	<.001	.001	<.001
**p-Tau217**					
Q2	−0.010 (−0.022 to 0.001)	−0.003 (−0.012 to 0.006)	−0.006 (−0.014 to 0.002)	−0.010 (−0.018 to −0.002)^c^	−0.008 (−0.014 to −0.001)^c^
Q3	−0.020 (−0.031 to −0.008)^d^	−0.019 (−0.028 to −0.011)^e^	−0.023 (−0.031 to −0.015)^e^	−0.021 (−0.030 to −0.013)^e^	−0.022 (−0.028 to −0.015)^e^
Q4 (highest)	−0.045 (−0.059 to −0.030)^e^	−0.034 (−0.045 to −0.023)^e^	−0.047 (−0.056 to −0.037)^e^	−0.035 (−0.045 to −0.025)^e^	−0.045 (−0.053 to −0.037)^e^
*P* value for trend	<.001	<.001	<.001	<.001	<.001
**p-Tau181**					
Q2	−0.012 (−0.023 to −0.000)^c^	−0.009 (−0.018 to −0.000)^c^	−0.006 (−0.014 to 0.002)	−0.008 (−0.016 to −0.000)^c^	−0.009 (−0.015 to −0.003)^d^
Q3	−0.024 (−0.036 to −0.012)^e^	−0.021 (−0.030 to −0.012)^e^	−0.026 (−0.034 to −0.018)^e^	−0.026 (−0.034 to −0.017)^e^	−0.026 (−0.032 to −0.019)^e^
Q4 (highest)	−0.040 (−0.054 to −0.026)^e^	−0.036 (−0.047 to −0.025)^e^	−0.041 (−0.051 to −0.032)^e^	−0.036 (−0.046 to −0.026)^e^	−0.043 (−0.051 to −0.035)^e^
*P* value for trend	<.001	<.001	<.001	<.001	<.001
**t-Tau**					
Q2	−0.007 (−0.019 to 0.005)	−0.005 (−0.014 to 0.004)	−0.007 (−0.016 to 0.001)	−0.004 (−0.012 to 0.005)	−0.005 (−0.012 to 0.001)
Q3	−0.013 (−0.025 to −0.001)^c^	−0.012 (−0.022 to −0.003)^c^	−0.016 (−0.024 to −0.007)^e^	−0.006 (−0.014 to 0.003)	−0.013 (−0.020 to −0.006)^e^
Q4 (highest)	−0.009 (−0.022 to 0.004)	−0.015 (−0.025 to −0.005)^d^	−0.010 (−0.019 to −0.001)^c^	−0.007 (−0.016 to 0.002)	−0.012 (−0.019 to −0.004)^d^
*P* value for trend	.08	.001	.001	.11	<.001
**NfL**					
Q2	−0.015 (−0.026 to −0.004)^d^	−0.007 (−0.015 to 0.001)	−0.011 (−0.019 to −0.004)^d^	−0.017 (−0.025 to −0.010)^e^	−0.012 (−0.018 to −0.006)^e^
Q3	−0.022 (−0.034 to −0.010)^e^	−0.020 (−0.028 to −0.011)^e^	−0.027 (−0.035 to −0.019)^e^	−0.026 (−0.034 to −0.018)^e^	−0.025 (−0.031 to −0.018)^e^
Q4 (highest)	−0.046 (−0.061 to −0.031)^e^	−0.062 (−0.074 to −0.051)^e^	−0.062 (−0.071 to −0.052)^e^	−0.053 (−0.064 to −0.042)^e^	−0.058 (−0.066 to −0.050)^e^
*P* value for trend	<.001	<.001	<.001	<.001	<.001
**GFAP**					
Q2	−0.006 (−0.018 to 0.005)	−0.004 (−0.013 to 0.005)	−0.004 (−0.012 to 0.003)	−0.003 (−0.011 to 0.005)	−0.004 (−0.011 to 0.002)
Q3	−0.012 (−0.024 to −0.000)^c^	−0.017 (−0.026 to −0.008)^e^	−0.017 (−0.025 to −0.009)^e^	−0.015 (−0.023 to −0.006)^d^	−0.016 (−0.023 to −0.009)^e^
Q4 (highest)	−0.033 (−0.046 to −0.019)^e^	−0.038 (−0.048 to −0.027)^e^	−0.044 (−0.053 to −0.035)^e^	−0.036 (−0.046 to −0.026)^e^	−0.042 (−0.049 to −0.034)^e^
*P* value for trend	<.001	<.001	<.001	<.001	<.001

Abbreviations: Aβ, amyloid β; GFAP, glial fibrillary acidic protein; NfL, neurofilament light chain; p-tau, phosphorylated tau; Q, quartile; t-tau, total tau.

^a^ Beta coefficients with 95% CIs represent differences in annual rate of decline compared with the reference quartile, indicating the lowest level of brain pathology. All models were controlled for age, sex, education, anemia, kidney disease, obesity, cerebrovascular disease, and cardiovascular disease.

^b^ For the Aβ42/Aβ40 ratio, quartiles are presented in reverse order compared with the other biomarkers (Q1, lowest ratio and highest amyloid burden; Q4, highest ratio and lowest amyloid burden [reference]) to facilitate comparisons across biomarkers. For all other biomarkers, Q1 is the reference.

^c^ *P* < .05.

^d^ *P < *.01.

^e^ *P* < .001.

To explore the extent to which the association between AD-related biomarkers and cognitive decline was due to AD-specific or more general neurodegenerative influences, we reran the analyses with an AD-specific (p-tau217) and non–AD-specific (NfL or GFAP) marker in the same model (Table 3). In the analysis including p-tau217 and NfL, associations for both biomarkers remained significant but were attenuated compared with the single-biomarker models, with relatively larger effect sizes observed for NfL (global cognition Q4: β = −0.047 [95% CI, −0.056 to −0.038] vs −0.029 [95% CI, −0.037 to −0.020] for p-tau217). The analysis including p-tau217 and GFAP showed a similar pattern, although effect sizes tended to be relatively larger for p-tau217 (global cognition Q4: β = −0.036 [95% CI, −0.044 to −0.028] vs −0.032 [95% CI, −0.040 to −0.024]).

**Table 3.** Estimates From Linear Mixed Models by Quartiles of p-Tau217 and NfL or GFAP (N = 2008), Entered Simultaneously in the Same Model, for 4 Specific Cognitive Domains and Global Cognition

Biomarker	Cognitive domain, β (95% CI)^a^				Global cognition, β (95% CI)^a^
	Episodic memory	Semantic memory	Verbal fluency	Perceptual speed	
**p-Tau217 and NfL**					
p-tau217					
Q2^b^	−0.009 (−0.021 to 0.002)	−0.002 (−0.011 to 0.007)	−0.004 (−0.012 to 0.003)	−0.008 (−0.016 to −0.000)^c^	−0.006 (−0.012 to 0.001)
Q3	−0.014 (−0.026 to −0.003)^c^	−0.013 (−0.021 to −0.004)^d^	−0.015 (−0.023 to −0.008)^e^	−0.015 (−0.023 to −0.007)^e^	−0.015 (−0.021 to −0.008)^e^
Q4 (highest)	−0.034 (−0.049 to −0.019)^e^	−0.017 (−0.028 to −0.006)^d^	−0.029 (−0.039 to −0.019)^e^	−0.020 (−0.031 to −0.009)^e^	−0.029 (−0.037 to −0.020)^e^
NfL					
Q2^b^	−0.013 (−0.024 to −0.002)^c^	−0.005 (−0.014 to 0.003)	−0.009 (−0.017 to −0.002)^c^	−0.016 (−0.023 to −0.008)^e^	−0.010 (−0.016 to −0.004)^d^
Q3	−0.016 (−0.028 to −0.003)^c^	−0.015 (−0.024 to −0.006)^d^	−0.021 (−0.029 to −0.013)^e^	−0.021 (−0.030 to −0.013)^e^	−0.018 (−0.025 to −0.012)^e^
Q4 (highest)	−0.035 (−0.051 to −0.019)^e^	−0.056 (−0.068 to −0.043)^e^	−0.050 (−0.061 to −0.040)^e^	−0.045 (−0.057 to −0.034)^e^	−0.047 (−0.056 to −0.038)^e^
**p-Tau217 and GFAP**					
p-Tau217					
Q2^b^	−0.010 (−0.021 to 0.002)	−0.002 (−0.011 to 0.007)	−0.004 (−0.012 to 0.003)	−0.008 (−0.016 to −0.000)^c^	−0.006 (−0.012 to 0.000)
Q3	−0.016 (−0.028 to −0.005)^d^	−0.015 (−0.024 to −0.006)^d^	−0.018 (−0.026 to −0.010)^e^	−0.017 (−0.026 to −0.009)^e^	−0.017 (−0.023 to −0.010)^e^
Q4 (highest)	−0.039 (−0.054 to −0.024)^e^	−0.027 (−0.038 to −0.015)^e^	−0.037 (−0.047 to −0.027)^e^	−0.027 (−0.037 to −0.017)^e^	−0.036 (−0.044 to −0.028)^e^
GFAP					
Q2^b^	−0.006 (−0.017 to 0.006)	−0.003 (−0.012 to 0.006)	−0.004 (−0.011 to 0.004)	−0.003 (−0.010 to 0.005)	−0.004 (−0.010 to 0.002)
Q3	−0.008 (−0.020 to 0.004)	−0.013 (−0.023 to −0.004)^d^	−0.012 (−0.020 to −0.004)^d^	−0.011 (−0.020 to −0.003)^d^	−0.012 (−0.018 to −0.005)^d^
Q4 (highest)	−0.024 (−0.038 to −0.010)^d^	−0.031 (−0.042 to −0.020)^e^	−0.034 (−0.043 to −0.025)^e^	−0.029 (−0.039 to −0.019)^e^	−0.032 (−0.040 to −0.024)^e^

Abbreviations: GFAP, glial fibrillary acidic protein; NfL, neurofilament light chain; p-tau, phosphorylated tau; Q, quartile.

^a^ Beta coefficients together with 95% CIs represent differences in annual rate of decline compared with the reference quartile, indicating the lowest level of brain pathology. All models were controlled for age, sex, education, anemia, kidney disease, obesity, cerebrovascular disease, and cardiovascular disease.

^b^ Q1 is the reference group.

^c^ *P* < .05.

^d^ *P* < .01.

^e^ *P* < .001.

Results from the linear mixed models treating the biomarkers as continuous variables are presented in eTable 9 in Supplement 1. Results were consistent with the quartile analyses, and lower Aβ42/Aβ40 ratio (β = 0.008 [95% CI, 0.005-0.010]) and higher concentrations of p-tau217 (β = −0.036 [95% CI, −0.042 to −0.031]) p-tau181 (β = −0.024 [95% CI, −0.029 to −0.020]), t-tau (β = −0.005 [95% CI, −0.009 to −0.002]), NfL (β = −0.015 [95% CI, −0.019 to −0.012]), and GFAP (β = −0.008 [95% CI, −0.011 to −0.005]) were associated with faster cognitive decline.

To further explore whether the associations for NfL and GFAP were dependent on level of AD-specific pathology, we applied a cutoff for p-tau217 representing low vs high risk of developing dementia in the SNAC-K cohort.^22^ Significant interactions were observed between p-tau217 (low vs high) and NfL for level (β = −0.068 [95% CI, −0.133 to −0.004]; *P* = .04) and change (β = 0.012 [95% CI, 0.005-0.020]; *P* = .002), reflecting that higher levels of NfL were associated with relatively worse cognitive performance, whereas the association between NfL and rate of change was relatively smaller within the high–p-tau217 group. Similarly, the association with GFAP and rate of change was relatively smaller (β interaction = 0.013 [95% CI, 0.006-0.019]; *P* < .001) and nonsignificant in the high–p-tau217 group. The β values for associations with level and change in global cognition for NfL and GFAP in the total sample and the low– and high–p-tau217 groups are presented in eTable 10 in Supplement 1.

It is a noteworthy pattern that for p-tau217, β values for episodic memory and verbal fluency tended to be relatively larger, whereas for NfL, β values for episodic memory were relatively smaller compared with the other domains in the results reported in Table 2, Table 3, and eTable 9 in Supplement 1. We ran bootstrapping analyses comparing the β values for rate of change in specific domains from eTable 9 in Supplement 1 for p-tau217 and NfL. Bootstrapping with 500 and 600 resamples yielded consistent results. Across the 6 comparisons, estimated differences were small and confidence intervals included 0 for most contrasts. The exception was the comparison between episodic memory and verbal fluency for NfL, showing a significantly smaller effect size (*P* = .02) with episodic memory decline (β = −0.011 [95% CI, −0.017 to −0.004]; *P* = .001) vs verbal fluency decline (β = −0.017 [95% CI, −0.021 to −0.012]; *P* < .001).

### Interactions With *APOE*, Age, and Sex

We checked for 3-way interactions for global cognition in the analyses using the biomarkers as continuous variables. Significant interactions were observed between *APOE* and p-tau181 (β = −0.016 [95% CI, −0.027 to −0.006], NfL (β = 0.021 [95% CI, 0.013-0.028]), and GFAP (β = 0.014 [95% CI, 0.007-0.020]) and between sex and NfL (β = −0.014 [95% CI, −0.021 to −0.007]) and GFAP (β = −0.017 [95% CI, −0.025 to −0.010]) (Table 4). The association between higher levels of p-tau181 and faster cognitive decline was almost twice as large in ε4 carriers vs noncarriers (β = −0.037 [95% CI, −0.047 to −0.026] vs −0.020 [95% CI, −0.025 to −0.015], respectively). Positive 3-way interactions were observed for NfL and GFAP, reflecting no associations in ε4 carriers. Moreover, associations between higher levels of NfL and GFAP and faster rates of cognitive decline were exacerbated in females (β = −0.023 [95% CI, −0.029 to −0.018] and −0.021 [95% CI, −0.028 to −0.015], respectively) (both *P* < .001) (full stratified analyses are provided in eTables 11-16 in Supplement 1).

**Table 4.** Three-Way Interactions Among Blood Biomarkers of Alzheimer Disease, Time, and Age; Sex; or *APOE* Genotype for Global Cognition

Biomarker	Estimate, β (95% CI)		
	Age (<78 vs ≥78 y)	Sex (male vs female)	*APOE* (ε4 noncarriers vs ε4 carriers)
Aβ42/Aβ40	0.002 (−0.005 to 0.009)	0.000 (−0.006 to 0.006)	0.005 (−0.003 to 0.012)
p-Tau217	−0.010 (−0.022 to 0.003)	−0.004 (−0.015 to 0.008)	−0.010 (−0.023 to 0.002)
p-Tau181	−0.010 (−0.020 to 0.001)	−0.005 (−0.014 to 0.005)	−0.016 (−0.027 to −0.006)^a^
t-Tau	−0.001 (−0.008 to 0.007)	−0.006 (−0.012 to 0.000)	0.006 (−0.000 to 0.012)
NfL	−0.005 (−0.013 to 0.003)	−0.014 (−0.021 to −0.007)^b^	0.021 (0.013 to 0.028)^b^
GFAP	−0.006 (−0.014 to 0.002)	−0.017 (−0.025 to −0.010)^b^	0.014 (0.007 to 0.020)^b^

Abbreviations: Aβ, amyloid β; GFAP, glial fibrillary acidic protein; NfL, neurofilament light chain; p-tau, phosphorylated tau; t-tau, total tau.

^a^ *P* < .01.

^b^ *P* < .001.

## Discussion

This cohort study found that levels of AD blood biomarkers, especially being in the quartile implying the highest level of brain pathology, were associated with faster rates of cognitive decline in a sample of older adults with up to 15 years of follow-up. The associations were most evident for p-tau217, p-tau181, NfL, and GFAP. The pattern of results for these biomarkers suggests a global impact on cognitive aging, with some variations observed across cognitive domains.

The most notable associations with cognitive decline were observed for the 2 p-tau isoforms, which are in line with previous studies on blood-based AD biomarkers showing the most consistent associations with p-tau.^13^ Similarly, studies assessing tau pathology with positron emission tomography (PET) or cerebrospinal fluid have found strong correlations with cognitive performance.^29,30^ Blood-derived p-tau217 has displayed comparable associations with cognitive decline as PET-derived AD markers and has been shown to contribute significantly to the prediction of cognitive decline over and above the PET markers.^6,16,31^ Phosphorylated tau 217 is believed to reflect amyloid as well as tau burden in the brain^32^ and, thus, may be well suited to capture very early cognitive effects of AD pathology. Consistent with previous studies favoring p-tau over t-tau as a peripheral blood marker of AD, the smallest effect sizes were observed for t-tau, a nonspecific marker of neuronal damage.^33,34^

We observed significant, although somewhat reduced, associations with the Aβ42/Aβ40 ratio. Other studies have shown associations with cognitive decline in individuals with normal cognition at baseline,^14,17^ although some observed no, or relatively small, associations.^18,20^ The smaller effect sizes for the Aβ42/Aβ40 ratio may reflect lower concentrations of Aβ in blood compared with cerebrospinal fluid, substantial peripheral production of Aβ, and reduced accuracy in quantifying blood levels of amyloid. In contrast, the presence of p-tau, specifically p-tau217, in blood is considered a core biomarker of AD, with a closer link to brain pathology.^6^

Neurofilament light chain is a nonspecific marker of loss of axonal integrity, and previous findings regarding rate of cognitive decline have been mixed. Significant associations have been shown in both cohorts without cognitive impairment and mixed-sample cohorts,^15,17,18,19,20,35,36,37^ although lack of such associations has also been observed.^3,14,38^ Studies including GFAP have reported similar effect sizes as NfL,^18,19,37^ although some studies found associations only for GFAP,^14,38,39^ suggesting that these markers may partly capture different underlying pathology. We observed relatively large effect sizes for NfL compared with GFAP. Moreover, including both p-tau217 and NfL in the same model revealed comparatively relatively more pronounced associations of NfL to cognitive decline compared with p-tau217. Neurofilament light chain has been associated with neurodegeneration in brain regions not typically affected in AD^40^ and small vessel disease burden indicative of poorer vascular brain health.^41,42^ A previous study observed associations between NfL and future dementia only in an amyloid-negative group, suggesting that NfL might be most valuable as a biomarker for non-AD dementia.^43^ Relatedly, we observed a more pronounced association with cognitive decline for individuals with low p-tau217. Similar but attenuated results were obtained for GFAP. These larger associations in the low–p-tau217 group suggest that non–AD-specific brain alterations may have exerted a stronger influence in this sample with lower AD pathologic burden and lower dementia risk. The relatively large effect sizes observed for NfL in our study suggest that it could be a useful marker of cognitive decline due to neuropathology beyond AD, which is commonly present in the general older population.^44,45^

A recent cross-sectional study observed a specific association of p-tau217 and p-tau181 with episodic memory, an early marker of AD, whereas NfL was associated with broader cognitive deficits.^11^ Similarly, in a sample of individuals with mixed cognitive status, p-tau217, but not NfL, was associated with episodic memory decline, especially in those with Aβ positivity.^12^ These observations partly align with our longitudinal findings in which the estimates for p-tau measures tended to be numerically higher for episodic memory decline, whereas effect sizes for NfL were relatively smaller for episodic memory compared with other domains. However, these differences were only statistically significant when comparing the estimates for episodic memory and verbal fluency for NfL. Of note, another longitudinal study found the strongest link between p-tau217 and semantic memory decline, especially in Black participants.^20^ Together, these results suggest some degree of specificity for AD-related domains for p-tau markers, although confirmation in larger samples is needed. In contrast, the broader associations across cognitive domains observed for NfL suggest that it may be suitable as a marker of AD-nonspecific axonal injury in the general older population.

Long follow-up periods may be necessary to capture associations between biomarkers and cognitive change, especially in adults with normal cognition. In line with this reasoning, Dubbelman et al^46^ found that most observed associations with rate of cognitive decline disappeared when follow-up time was limited to 3 years. Very early in dementia development, when biomarker levels are low, changes in cognitive decline trajectories are still subtle and may only be detected with fine-tuned neuropsychological testing that is sensitive to gradually accumulating brain pathology or reflected indirectly as a slower learning curve^47^ or lack of practice effects.^48^

We observed significant interactions with sex and genetic risk for AD, suggesting that these factors may play a role in the biomarker-cognition link. Associations between NfL and GFAP with cognitive decline were exacerbated in female participants. Higher dementia prevalence and higher GFAP levels in women may have contributed to this finding.^49,50^ Because these markers are not specific for AD-type dementia, other copathologies, which may affect women more severely than men, also may have influenced this association.^51,52^ In contrast, individuals with high levels of p-tau181 who also carry the *APOE* ε4 allele could be expected to be on the pathway toward a clinical manifestation of AD. Indeed, we found that the association between p-tau181 and cognitive decline was almost twice as large among ε4 carriers. Conversely, associations between higher levels of NfL and GFAP and cognitive decline only appeared in ε4 noncarriers, again suggesting that this decline may mostly reflect non-AD pathology. In contrast to our findings, a community-based study of Black and White older adults found that associations of t-tau, GFAP, and NfL with rate of cognitive decline were mainly observed in ε4 carriers.^15^ A large clinic-based cohort study instead did not find any modification of the association between blood-based biomarkers of Aβ, tau, or NfL and rates of cognitive decline by sex or *APOE*.^35^ Potential reasons for these inconsistencies may be differences in the biomarkers assessed (eg, the inclusion of p-tau) or sample characteristics (eg, race and ethnicity, cognitive status at baseline).

It should be noted that biomarker levels in this sample of individuals without dementia were generally low, and associations with cognitive decline were detected primarily for those with the highest biomarker concentrations. However, we observed a clear dose-response association for each quartile of p-tau217, p-tau181, and NfL, and the results were confirmed using the biomarkers as continuous variables. Clinically defined cutoffs may help identify at-risk individuals who might benefit from more extensive examinations. The differential patterns observed in this and other studies further suggest that distinct biomarker profiles may provide clues to different mechanistic processes for cognitive decline, with some associated more with AD and others associated more with non-AD or mixed dementia etiologies. These biomarker profiles may be used to support more individualized risk stratification and tailored interventions.

### Strengths and Limitations

Major strengths of this study included a comprehensive selection of biomarkers, including p-tau217; a large, well-characterized sample with long follow-up; and assessment of performance in specific cognitive domains. The study also had some limitations. First, it should be noted that the examinations were performed in a White cohort with relatively high socioeconomic status, which may limit the generalizability of the findings. Second, biomarker concentrations measured in serum tend to be lower than in other matrices and may be susceptible to peripheral contamination. They also preclude the application of clinical cutoffs that have been derived for plasma markers. The possibility to make mechanistic interpretations is furthermore limited by the absence of longitudinal biomarker data. Missing biomarker data were more common among older participants, women, and participants with lower education and baseline cognition. Moreover, individuals with higher biomarker concentrations generally stayed in the study for shorter periods, which potentially led to an underestimation of the true associations. Finally, including multiple outcomes increases the risk of type I errors; therefore, domain-specific results should be interpreted with some caution. Nevertheless, except for t-tau, applying a stricter *P* value (*P* < .01) for the specific domains to adjust for the number of outcomes, more than 95% of the associations for Q3 and Q4 remained significant.

## Conclusions

This cohort study found that elevated AD-related blood biomarkers, potentially reflecting different types of dementia-related brain pathology, are associated with faster subsequent cognitive decline, even among individuals without dementia in the general population. The modifying effects of sex and *APOE* underscore the need for individualized interpretation of the prognostic value of these biomarkers.

## Notes

Supplement 1. **eTable 1.** Lower Limit of Quantification (LLOQ)/Limit of Detection (LOD) and Intra- or Intercoefficient of Variation (% CV) for All Included Biomarkers **eTable 2. **Intra- and Interassay Precision Expressed as Coefficient of Variation (% CV) **eTable 3. **Baseline Characteristics of Study Participants With and Without Follow-Up Data **eTable 4. **Correlation Matrix Showing Spearman Correlations Between Blood Biomarkers of Alzheimer Disease and Cognitive Domains **eTable 5. **Estimates From Linear Mixed Models by Quartiles of 6 Individual Biomarkers of Alzheimer Disease (n = 1954) for 4 Specific Cognitive Domains and Global Cognition, Additionally Controlling for *APOE* Genotype **eTable 6. **Estimates From Linear Mixed Models by Quartiles of 6 Individual Biomarkers of Alzheimer Disease for 4 Specific Cognitive Domains and Global Cognition in Individuals with Mini-Mental State Examination Score Greater Than 26 (n = 1870) **eTable 7. **Estimates From Linear Mixed Models by Quartiles of 6 Individual Biomarkers of Alzheimer Disease for 4 Specific Cognitive Domains and Global Cognition in Participants With Available Follow-Up Data (n = 1481) **eTable 8. **Estimates From Linear Mixed Models by Quartiles of 6 Individual Biomarkers of Alzheimer Disease for Global Cognition in Participants With Data on All 4 Cognitive Domains (n = 1935) **eTable 9. **Estimates from Linear Mixed Models for 6 Continuous Individual Biomarkers of Alzheimer Disease in the Total Sample (N = 2008) for 4 Specific Cognitive Domains and Global Cognition **eTable 10. **Estimates from Linear Mixed Models for NfL and GFAP in the Total Sample (N = 2008) and in Individuals With Low (n = 1212) and High (n = 796) p-Tau217 for Global Cognition **eTable 11. **Estimates From Linear Mixed Models for 6 Continuous Individual Biomarkers of Alzheimer Disease in *APOE *ε4 Noncarriers (n = 1375) for 4 Specific Cognitive Domains and Global Cognition **eTable 12. **Estimates From Linear Mixed Models for 6 Continuous Individual Biomarkers of Alzheimer Disease in *APOE *ε4 Carriers (n = 579) for 4 Specific Cognitive Domains and Global Cognition **eTable 13. **Estimates From Linear Mixed Models for 6 Continuous Individual Biomarkers of Alzheimer Disease in Males (n = 793) for 4 Specific Cognitive Domains and Global Cognition **eTable 14.** Estimates From Linear Mixed Models for 6 Continuous Individual Biomarkers of Alzheimer Disease in Females (n = 1215) for 4 Specific Cognitive Domains and Global Cognition **eTable 15. **Estimates From Linear Mixed Models for 6 Continuous Individual Biomarkers of Alzheimer Disease in Young-Old (Aged <78 Years, n = 1294) for 4 Specific Cognitive Domains and Global Cognition **eTable 16.** Estimates From Linear Mixed Models for 6 Continuous Individual Biomarkers of Alzheimer Disease in Old-Old (Aged ≥78 Years, n = 714) for 4 Specific Cognitive Domains and Global Cognition **eFigure 1. **Flow Chart of Study Participation **eFigure 2. **Estimated Trajectories of Episodic Memory, Semantic Memory, Verbal Fluency, and Perceptual Speed Across 15 Years for Quartiles of 6 Blood-Based Biomarkers of Alzheimer Disease **eMethods 1. **Dementia Diagnosis **eMethods 2. **Classification of Diseases **eMethods 3.** Details on Protein Quantification

Supplement 2.
Data Sharing Statement
