# Supplementary material for: Blood Biomarkers of Alzheimer Disease and Rates of Global and Domain-Specific Cognitive Decline
Source: JAMA Netw Open. 2026 Aug 13;9(8):e2628975. doi: 10.1001/jamanetworkopen.2026.28975 (PMC13474032; doi:10.1001/jamanetworkopen.2026.28975)
Supplement: Supplementary file 1 [file jamanetwopen-e2628975-s001.pdf]

Supplemental Online Content

Laukka EJ, Ekström I, Valletta M, et al. Blood biomarkers of Alzheimer disease and rates of global and domain-specific cognitive decline. *JAMA Netw Open*. 2026;9(8):e2628975.
doi:10.1001/jamanetworkopen.2026.28975

eTable 1. Lower Limit of Quantification (LLOQ)/Limit of Detection (LOD) and Intra- or Intercoefficient of Variation (% CV) for All Included Biomarkers

eTable 2. Intra- and Interassay Precision Expressed as Coefficient of Variation (% CV)

eTable 3. Baseline Characteristics of Study Participants With and Without Follow-Up Data

eTable 4. Correlation Matrix Showing Spearman Correlations Between Blood Biomarkers of Alzheimer Disease and Cognitive Domains

eTable 5. Estimates From Linear Mixed Models by Quartiles of 6 Individual Biomarkers of Alzheimer Disease (n = 1954) for 4 Specific Cognitive Domains and Global Cognition, Additionally Controlling for *APOE* Genotype

eTable 6. Estimates From Linear Mixed Models by Quartiles of 6 Individual Biomarkers of Alzheimer Disease for 4 Specific Cognitive Domains and Global Cognition in Individuals with Mini-Mental State Examination Score Greater Than 26 (n = 1870)

eTable 7. Estimates From Linear Mixed Models by Quartiles of 6 Individual Biomarkers of Alzheimer Disease for 4 Specific Cognitive Domains and Global Cognition in Participants With Available Follow-Up Data (n = 1481)

eTable 8. Estimates From Linear Mixed Models by Quartiles of 6 Individual Biomarkers of Alzheimer Disease for Global Cognition in Participants With Data on All 4 Cognitive Domains (n = 1935)

eTable 9. Estimates from Linear Mixed Models for 6 Continuous Individual Biomarkers of Alzheimer Disease in the Total Sample (N = 2008) for 4 Specific Cognitive Domains and Global Cognition

eTable 10. Estimates from Linear Mixed Models for NfL and GFAP in the Total Sample (N = 2008) and in Individuals With Low (n = 1212) and High (n = 796) p-Tau217 for Global Cognition

eTable 11. Estimates From Linear Mixed Models for 6 Continuous Individual Biomarkers of Alzheimer Disease in *APOE* ε4 Noncarriers (n = 1375) for 4 Specific Cognitive Domains and Global Cognition

eTable 12. Estimates From Linear Mixed Models for 6 Continuous Individual Biomarkers of Alzheimer Disease in *APOE* ε4 Carriers (n = 579) for 4 Specific Cognitive Domains and Global Cognition

eTable 13. Estimates From Linear Mixed Models for 6 Continuous Individual Biomarkers of Alzheimer Disease in Males (n = 793) for 4 Specific Cognitive Domains and Global Cognition

eTable 14. Estimates From Linear Mixed Models for 6 Continuous Individual Biomarkers of Alzheimer Disease in Females (n = 1215) for 4 Specific Cognitive Domains and Global Cognition

eTable 15. Estimates From Linear Mixed Models for 6 Continuous Individual Biomarkers of Alzheimer Disease in Young-Old (Aged <78 Years, n = 1294) for 4 Specific Cognitive Domains and Global Cognition

eTable 16. Estimates From Linear Mixed Models for 6 Continuous Individual Biomarkers of Alzheimer Disease in Old-Old (Aged ≥78 Years, n = 714) for 4 Specific Cognitive Domains and Global Cognition

eFigure 1. Flow Chart of Study Participation

eFigure 2. Estimated Trajectories of Episodic Memory, Semantic Memory, Verbal Fluency, and Perceptual Speed Across 15 Years for Quartiles of 6 Blood-Based Biomarkers of Alzheimer Disease

© 2026 Laukka EJ et al. *JAMA Network Open*.

eMethods 1. Dementia Diagnosis

eMethods 2. Classification of Diseases

eMethods 3. Details on Protein Quantification

This supplemental material has been provided by the authors to give readers additional information about their work.

© 2026 Laukka EJ et al. *JAMA Network Open*.

eTable 1. Lower Limit of Quantification (LLOQ)/Limit of Detection (LOD) and intra/inter coefficient of variation (% CV) for all included biomarkers

Acronym	Biomarker	LLOQ/<br>LOD range (pg/mL)	Intra /Inter CV(%)	Reagents	Analytical platform
**Aβ40**	Amyloid beta protein 40	1.90<br>0.0550-0.490*	2.2/5.0	Neuro 3-plex A;101995;Lot 503659	Quanterix Simoa SR-X
**Aβ42**	Amyloid beta protein 42	0.225<br>0.0714-0.201*	4.8/7.1	Neuro 3-plex A;101995;Lot 503659	Quanterix Simoa SR-X
**P-tau217**	phosphorylated tau 217	0.004<br>0.0003-0.0004**	11/13.4	ALZpath p-Tau 217 Dvantage plus, Lot 504307	Quanterix Simoa SR-X
**P-tau181**	phosphorylated tau 181	0.085<br>0.018–0.060*	9.2/14.4	pTau-181 Advantage V2, Lot 503703	Quanterix Simoa SR-X
**T-tau**	Total tau	0.0715<br>0.0141-0.0194*	10.7/11.9	Neuro 3-plex A;101995;Lot 503659	Quanterix Simoa SR-X
**GFAP**	Glial fibrillary acidic protein	4.15<br>0.133-0.740*	13.1/21.4	Neuro 2-plex B; 103520; Lot 503409	Quanterix Simoa SR-X
**NfL**	Neurofilament light chain	0.400<br>0.012-0.149*	7.6/11.6	Neuro 2-plex B; 103520; Lot 503409	Quanterix Simoa SR-X

*LOQ and LOD specified in the product datasheet

**LOD and LLOQ calculated during assay validation and compatibility in house (Grande G, Valletta M, Rizzuto D, Xia X, Qiu C, Orsini N, Dale M, Andersson S, Fredolini C, Winblad B, Laukka EJ, Fratiglioni L, Vetrano DL. Blood-based biomarkers of Alzheimer's disease and incident dementia in the community. Nat Med. 2025 Jun;31(6):2027-2035).

LOD and LLOQ reported in the data sheets of each kit are calculated on a number of replicate measurements across different Lots and instruments. However, empirical LOD and LLOQ values are calculated for each plate analyzed and may vary slightly with each experiment and Lot. Empirical LOD and LOQ for each run are not reported in the output file. The instrument software calculates the concentrations according to the calibration curve analyzed in each plate together with the samples and returns NaN values when samples average number of enzymes per bead (AEB) are below or above calculated quantification limit.

© 2026 Laukka EJ et al. *JAMA Network Open*.

eTable 2. Intra- and Interassay Precision Expressed as Coefficient of Variation (% CV)

	Sample pool			Control 1			Control 2		
	**Conc**	**Within run**	**Between run**	**Conc**	**Within run**	**Between run**	**Conc**	**Within run**	**Between run**
**Aβ-40**	120.4	2.2	5.2	10.1	3.8	6.8	156.1	1.6	3.7
**Aβ-42**	6.6	4.8	7.4	3.4	6.1	8.2	76.8	2.1	4.7
**P-tau 217**	0.14	13.3	17.6	0.8	8.8	12.0	7.7	6.1	11.2
**P-tau 181**	1.5	9.2	13.6	3.6	4.9	7.9	98.0	3.2	5.9
**T-tau**	0.9	10.7	12.3	1.6	5.3	8.5	61.7	2.0	5.2
**NfL**	24.1	7.6	11.6	4.1	11.5	17.6	409.8	4.5	7.4
**GFAP**	194	13.1	21.4	89.8	11.4	17.4	10311	5.8	9.7

Conc: average of the concentrations (pg/mL) obtained in 39 runs.
Within run: CV (%) calculated on the triplicate pool, control 1 and control 2 included in each run (plate). The average CV for all runs is reported.
Between run: CV (%) calculated on the concentration values obtained for pool, control 1 and control 2 across all the runs (plate).
Ref: Grande G, Valletta M, Rizzuto D, Xia X, Qiu C, Orsini N, Dale M, Andersson S, Fredolini C, Winblad B, Laukka EJ, Fratiglioni L, Vetrano DL. Blood-based biomarkers of Alzheimer's disease and incident dementia in the community. Nat Med. 2025 Jun;31(6):2027-2035.

© 2026 Laukka EJ et al. *JAMA Network Open*.

eTable 3. Baseline Characteristics of Study Participants With and Without Follow-Up Data

Characteristic	Follow-up data (n=1481)	No follow-up data (n=527)
Age, y, mean (SD)	70.53 (9.47)	75.17 (10.98)
Female, n (%)	914 (61.72)	301 (57.12)
Education, y, mean (SD)	12.65 (4.23)	11.26 (4.07)
MMSE, mean (SD)	29.08 (1.12)	28.39 (1.67)
Anemia, n (%)	110 (7.43)	85 (16.13)
Kidney disease, n (%)	413 (27.89)	206 (39.09)
Obesity, n (%)	210 (14.18)	65 (12.33)
Heart disease, n (%)	250 (16.88)	159 (30.17)
Cerebrovascular disease, n (%)	63 (4.25)	38 (7.21)
Laboratory assessment		
Aβ42/Aβ40 ratio (median [IQR])	0.06 [0.05; 0.07]	0.06 [0.05; 0.06]
P-tau217 (pg/ml; median [IQR])	0.09 [0.05; 0.15]	0.13 [0.07; 0.26]
P-tau181 (pg/ml; median [IQR])	1.07 [0.70; 1.62]	1.44 [0.86; 2.26]
T-tau (pg/ml; median [IQR])	0.81 [0.53; 1.11]	0.95 [0.60; 1.39]
NfL (pg/ml; median [IQR])	16.19 [11.85; 23.94]	21.90 [14.40; 35.42]
GFAP (pg/ml; median [IQR])	109.38 [73.54; 166.16]	139.23 [94.20; 218.30]
APOE ε4 carrier, n (%)	425 (28.87)	154 (31.95)
Cognitive assessment		
Word recall, mean (SD)	7.35 (2.32)	6.34 (2.46)
Word recognition, mean (SD)	11.83 (2.74)	11.10 (3.26)
Vocabulary, mean (SD)	23.47 (4.60)	21.27 (5.80)
Letter F, mean (SD)	15.82 (5.35)	13.71 (5.57)
Letter A, mean (SD)	13.07 (4.93)	11.10 (5.19)
Category animals, mean (SD)	22.06 (6.33)	19.24 (6.70)
Category professions, mean (SD)	16.71 (5.33)	14.61 (5.68)
Digit cancellation, mean (SD)	18.14 (4.20)	16.24 (4.38)
Pattern comparison, mean (SD)	14.59 (3.76)	12.54 (4.22)

MMSE, Mini-Mental State Examination

© 2026 Laukka EJ et al. *JAMA Network Open*.

eTable 4. Correlation Matrix Showing Spearman Correlations Between Blood Biomarkers of Alzheimer Disease and Cognitive Domains

	Aβ42/Aβ40	P-tau217	P-tau181	T-tau	NfL	GFAP	EM	SM	VF	PS	Global
**Aβ42/Aβ40**	**1.00**	-0.42**	-0.41**	-0.34**	-0.20**	0.17**	0.13**	0.11**	0.13**	0.20**	0.19**
**P-tau217**	-0.42**	**1.00**	0.86***	0.62**	0.54**	0.43**	-0.21**	-0.18**	-0.21**	-0.35**	-0.32**
**P-tau181**	-0.41**	0.86***	**1.00**	0.60**	0.52**	0.39**	-0.19**	-0.20**	-0.21**	-0.33**	-0.31**
**T-tau**	-0.34**	0.62**	0.60**	**1.00**	0.28**	0.22**	-0.06**	-0.06*	-0.10**	-0.15**	-0.12**
**NfL**	-0.20**	0.54**	0.52**	0.28**	**1.00**	0.64**	-0.24**	-0.24**	-0.32**	0.52**	-0.44**
**GFAP**	-0.17**	0.43**	0.39**	0.22**	0.64**	**1.00**	-0.17**	-0.21**	-0.27**	-0.40**	-0.35**
**EM**	0.13**	-0.21**	-0.19**	-0.06**	-0.24**	-0.17**	**1.00**	0.34**	0.38**	0.34**	0.67**
**SM**	0.11**	-0.18**	-0.20**	0.06*	-0.24**	-0.21**	0.34**	**1.00**	0.56**	0.37**	0.74***
**VF**	0.13**	-0.21**	-0.21**	-0.10**	-0.32**	-0.27**	0.38**	0.56**	**1.00**	0.52**	0.81***
**PS**	0.20**	-0.35**	-0.33**	-0.15**	-0.52**	-0.40**	0.34**	0.37**	0.52**	**1.00**	0.75***
**Global**	0.19**	-0.32**	-0.31**	-0.12**	-0.44**	-0.35**	0.67**	0.74***	0.81***	0.75***	**1.00**

EM, episodic memory; SM, semantic memory; VF, verbal fluency; PS, perceptual speed
* $P< 0.05$, ** $<P< 0.01$, *** $P< 0.001$

© 2026 Laukka EJ et al. *JAMA Network Open*.

eTable 5. Estimates From Linear Mixed Models by Quartiles of 6 Individual Biomarkers of Alzheimer Disease (n = 1954) for 4 Specific Cognitive Domains and Global Cognition, Additionally Controlling for *APOE* Genotype. Beta coefficients together with 95% confidence intervals represent differences in annual rate of decline compared to the reference quartile, indicating the lowest level of brain pathology. All models were controlled for age, sex, education, anemia, kidney disease, obesity, cerebrovascular disease, cardiovascular disease, and *APOE* genotype.

		Episodic memory	Semantic memory	Verbal fluency	Perceptual speed	Global cognition
**Aβ42/Aβ40**	Q3[a]	-0.013 (-0.025 to -0.001)*	-0.009 (-0.018 to 0.000)	-0.005 (-0.013 to 0.003)	-0.006 (-0.015 to 0.002)	-0.009 (-0.016 to -0.002)*
	Q2	-0.009 (-0.022 to 0.003)	-0.018 (-0.028 to -0.009)***	-0.012 (-0.021 to -0.004)**	-0.010 (-0.019 to -0.001)*	-0.013 (-0.020 to -0.006)***
	Q1	-0.021 (-0.034 to -0.009)**	-0.017 (-0.027 to -0.007)**	-0.017 (-0.026 to -0.008)***	-0.016 (-0.025 to -0.007)**	-0.019 (-0.026 to -0.012)***
P trend		< 0.01	< 0.001	< 0.001	< 0.001	< 0.001
**P-tau217**	Q2	-0.011 (-0.023 to 0.000)	-0.003 (-0.012 to 0.006)	-0.006 (-0.014 to 0.001)	-0.010 (-0.018 to -0.002)*	-0.008 (-0.014 to -0.001)*
	Q3	-0.020 (-0.031 to -0.008)**	-0.020 (-0.029 to -0.011)***	-0.023 (-0.031 to -0.016)***	-0.022 (-0.030 to -0.013)***	-0.022 (-0.029 to -0.016)***
	Q4	-0.046 (-0.060 to -0.031)***	-0.034 (-0.045 to -0.023)***	-0.047 (-0.056 to -0.037)***	-0.035 (-0.045 to -0.025)***	-0.045 (-0.053 to -0.037)***
P trend		< 0.001	< 0.001	< 0.001	< 0.001	< 0.001
**P-tau181**	Q2	-0.011 (-0.023 to -0.000)	-0.010 (-0.018 to -0.001)*	-0.007 (-0.014 to 0.001)	-0.009 (-0.017 to -0.000)*	-0.009 (-0.016 to -0.003)**
	Q3	-0.024 (-0.035 to -0.012)***	-0.021 (-0.030 to -0.012)***	-0.026 (-0.034 to -0.018)***	-0.026 (-0.034 to -0.018)***	-0.026 (-0.032 to -0.019)***
	Q4	-0.040 (-0.054 to -0.026)***	-0.037 (-0.047 to -0.026)***	-0.042 (-0.051 to -0.032)***	-0.037 (-0.047 to -0.027)***	-0.043 (-0.051 to -0.035)***
P trend		< 0.001	< 0.001	< 0.001	< 0.001	< 0.001
**T-tau**	Q2	-0.007 (-0.019 to 0.005)	-0.006 (-0.015 to 0.003)	-0.008 (-0.016 to 0.001)	-0.004 (-0.013 to 0.005)	-0.006 (-0.013 to 0.001)
	Q3	-0.013 (-0.025 to -0.000)*	-0.012 (-0.021 to -0.002)*	-0.015 (-0.024 to -0.006)**	-0.005 (-0.014 to 0.004)	-0.013 (-0.020 to -0.006)***
	Q4	-0.009 (-0.022 to 0.003)	-0.015 (-0.025 to -0.005)**	-0.011 (-0.020 to -0.002)*	-0.008 (-0.017 to 0.001)	-0.012 (-0.020 to -0.005)**
P trend		n.s.	< 0.01	< 0.01	n.s.	< 0.001
**NfL**	Q2	-0.016 (-0.027 to -0.004)**	-0.007 (-0.015 to 0.002)	-0.012 (-0.019 to -0.004)**	-0.017 (-0.025 to -0.010)***	-0.012 (-0.018 to -0.006)***
	Q3	-0.021 (-0.033 to -0.009)***	-0.020 (-0.028 to -0.011)***	-0.027 (-0.035 to -0.019)***	-0.026 (-0.034 to -0.018)***	-0.024 (-0.031 to -0.018)***
	Q4	-0.046 (-0.061 to -0.031)***	-0.063 (-0.075 to -0.052)***	-0.061 (-0.071 to -0.052)***	-0.053 (-0.064 to -0.042)***	-0.058 (-0.066 to -0.050)***
P trend		< 0.001	< 0.001	< 0.001	< 0.001	< 0.001
**GFAP**	Q2	-0.006 (-0.017 to 0.006)	-0.004 (-0.013 to 0.005)	-0.005 (-0.013 to 0.003)	-0.003 (-0.012 to 0.005)	-0.004 (-0.011 to 0.002)
	Q3	-0.011 (-0.023 to 0.001)	-0.016 (-0.025 to -0.007)**	-0.017 (-0.025 to -0.008)***	-0.014 (-0.023 to -0.006)**	-0.016 (-0.022 to -0.009)***
	Q4	-0.033 (-0.047 to -0.020)***	-0.039 (-0.050 to -0.028)***	-0.044 (-0.054 to -0.035)***	-0.036 (-0.046 to -0.026)***	-0.042 (-0.050 to -0.035)***
P trend		< 0.001	< 0.001	< 0.001	< 0.001	< 0.001

[a] For the Aβ42/Aβ40 ratio, quartiles are presented in reverse order relative to other the biomarkers (Q1 = lowest ratio/highest amyloid burden; Q4 = highest ratio/lowest amyloid burden [reference]) to facilitate comparisons across biomarkers. For all other biomarkers, Q1 is the reference.
* *P*< 0.05, ** *P*< 0.01, *** *P*< 0.001

© 2026 Laukka EJ et al. *JAMA Network Open*.

eTable 6. Estimates From Linear Mixed Models by Quartiles of 6 Individual Biomarkers of Alzheimer Disease for 4 Specific Cognitive Domains and Global Cognition in Individuals with Mini-Mental State Examination Score Greater Than 26 (n = 1870). Beta coefficients together with 95% confidence intervals represent differences in annual rate of decline compared to the reference quartile, indicating the lowest level of brain pathology. All models were controlled for age, sex, education, anemia, kidney disease, obesity, cerebrovascular disease, and cardiovascular disease.

		Episodic memory	Semantic memory	Verbal fluency	Perceptual speed	Global cognition
**Aβ42/Aβ40**	Q3[a]	-0.013 (-0.026 to -0.000)*	-0.011 (-0.021 to -0.001)*	-0.004 (-0.013 to 0.004)	-0.005 (-0.014 to 0.004)	-0.009 (-0.016 to -0.002)*
	Q2	-0.006 (-0.019 to 0.007)	-0.020 (-0.030 to -0.010)***	-0.011 (-0.019 to -0.002)*	-0.008 (-0.017 to 0.001)	-0.012 (-0.019 to -0.004)**
	Q1	-0.026 (-0.039 to -0.012)***	-0.018 (-0.029 to -0.008)**	-0.019 (-0.028 to -0.010)***	-0.016 (-0.026 to -0.007)**	-0.021 (-0.029 to -0.013)***
P trend		< 0.01	< 0.001	< 0.001	< 0.01	< 0.001
**P-tau217**	Q2	-0.011 (-0.023 to 0.002)	-0.005 (-0.014 to 0.005)	-0.007 (-0.015 to 0.001)	-0.012 (-0.021 to -0.004)**	-0.009 (-0.016 to -0.002)*
	Q3	-0.025 (-0.037 to -0.012)***	-0.017 (-0.026 to -0.008)***	-0.025 (-0.033 to -0.016)***	-0.023 (-0.032 to -0.015)***	-0.022 (-0.029 to -0.016)***
	Q4	-0.046 (-0.061 to -0.032)***	-0.039 (-0.051 to -0.028)***	-0.048 (-0.057 to -0.038)***	-0.037 (-0.047 to -0.027)***	-0.046 (-0.054 to -0.038)***
P trend		< 0.001	< 0.001	< 0.001	< 0.001	< 0.001
**P-tau181**	Q2	-0.014 (-0.026 to -0.002)*	-0.010 (-0.019 to -0.001)*	-0.006 (-0.015 to 0.002)	-0.008 (-0.017 to 0.000)	-0.010 (-0.017 to -0.003)**
	Q3	-0.024 (-0.036 to -0.011)***	-0.020 (-0.030 to -0.011)***	-0.023 (-0.031 to -0.014)***	-0.025 (-0.034 to -0.017)***	-0.024 (-0.031 to -0.017)***
	Q4	-0.043 (-0.058 to -0.029)***	-0.037 (-0.049 to -0.026)***	-0.041 (-0.050 to -0.031)***	-0.037 (-0.047 to -0.027)***	-0.043 (-0.051 to -0.035)***
P trend		< 0.001	< 0.001	< 0.001	< 0.001	< 0.001
**T-tau**	Q2	-0.009 (-0.022 to 0.004)	-0.005 (-0.015 to 0.005)	-0.009 (-0.018 to -0.000)*	-0.004 (-0.013 to 0.005)	-0.007 (-0.014 to 0.001)
	Q3	-0.015 (-0.028 to -0.002)*	-0.012 (-0.022 to -0.002)*	-0.016 (-0.025 to -0.007)***	-0.004 (-0.013 to 0.005)	-0.013 (-0.020 to -0.005)**
	Q4	-0.013 (-0.027 to 0.000)	-0.017 (-0.027 to -0.007)**	-0.012 (-0.021 to -0.003)**	-0.010 (-0.020 to -0.000)*	-0.014 (-0.022 to -0.007)***
P trend		< 0.05	< 0.01	< 0.01	n.s.	< 0.001
**NfL**	Q2	-0.018 (-0.030 to -0.006)**	-0.004 (-0.013 to 0.005)	-0.009 (-0.017 to -0.001)*	-0.014 (-0.023 to -0.006)**	-0.011 (-0.017 to -0.004)**
	Q3	-0.022 (-0.035 to -0.010)***	-0.019 (-0.028 to -0.009)***	-0.025 (-0.033 to -0.016)***	-0.026 (-0.035 to -0.017)***	-0.023 (-0.030 to -0.016)***
	Q4	-0.048 (-0.063 to -0.032)***	-0.059 (-0.071 to -0.047)***	-0.060 (-0.070 to -0.050)***	-0.050 (-0.061 to -0.039)***	-0.055 (-0.064 to -0.047)***
P trend		< 0.001	< 0.001	< 0.001	< 0.001	< 0.001
**GFAP**	Q2	-0.001 (-0.013 to 0.012)	-0.001 (-0.010 to 0.009)	-0.002 (-0.011 to 0.006)	-0.002 (-0.011 to 0.006)	-0.001 (-0.008 to 0.006)
	Q3	-0.013 (-0.025 to 0.000)	-0.016 (-0.026 to -0.007)**	-0.016 (-0.025 to -0.008)***	-0.015 (-0.024 to -0.006)**	-0.016 (-0.023 to -0.009)***
	Q4	-0.034 (-0.048 to -0.020)***	-0.036 (-0.047 to -0.025)***	-0.043 (-0.052 to -0.033)***	-0.035 (-0.045 to -0.025)***	-0.040 (-0.048 to -0.032)***
P trend		< 0.001	< 0.001	< 0.001	< 0.001	< 0.001

[a] For the Aβ42/Aβ40 ratio, quartiles are presented in reverse order relative to other the biomarkers (Q1 = lowest ratio/highest amyloid burden; Q4 = highest ratio/lowest amyloid burden [reference]) to facilitate comparisons across biomarkers. For all other biomarkers, Q1 is the reference.
* $P< 0.05$, ** $P< 0.01$, *** $P< 0.001$

© 2026 Laukka EJ et al. *JAMA Network Open*.

eTable 7. Estimates From Linear Mixed Models by Quartiles of 6 Individual Biomarkers of Alzheimer Disease for 4 Specific Cognitive Domains and Global Cognition in Participants With Available Follow-Up Data (n = 1481). Beta coefficients together with 95% confidence intervals represent differences in annual rate of decline compared to the reference quartile, indicating the lowest level of brain pathology. All models were controlled for age, sex, education, anemia, kidney disease, obesity, cerebrovascular disease, and cardiovascular disease.

		Episodic memory	Semantic memory	Verbal fluency	Perceptual speed	Global cognition
**Aβ42/Aβ40**	Q3[a]	-0.013 (-0.026 to -0.000)*	-0.010 (-0.020 to -0.000)*	-0.005 (-0.014 to 0.004)	-0.007 (-0.016 to 0.003)	-0.009 (-0.017 to -0.002)*
	Q2	-0.005 (-0.018 to 0.008)	-0.021 (-0.031 to -0.010)***	-0.012 (-0.021 to -0.003)*	-0.009 (-0.018 to 0.001)	-0.012 (-0.020 to -0.005)**
	Q1	-0.026 (-0.039 to -0.012)***	-0.018 (-0.029 to -0.008)**	-0.019 (-0.028 to -0.010)***	-0.017 (-0.027 to -0.008)***	-0.022 (-0.029 to -0.014)***
P trend		< 0.01	< 0.001	< 0.001	< 0.001	< 0.001
**P-tau217**	Q2	-0.013 (-0.026 to -0.000)*	-0.003 (-0.013 to 0.007)	-0.007 (-0.016 to 0.001)	-0.011 (-0.020 to -0.002)*	-0.009 (-0.016 to -0.002)*
	Q3	-0.024 (-0.037 to -0.012)***	-0.021 (-0.031 to -0.011)***	-0.027 (-0.036 to -0.019)***	-0.025 (-0.034 to -0.016)***	-0.025 (-0.032 to -0.018)***
	Q4	-0.045 (-0.059 to -0.030)***	-0.035 (-0.046 to -0.023)***	-0.047 (-0.057 to -0.038)***	-0.036 (-0.046 to -0.026)***	-0.045 (-0.053 to -0.037)***
P trend		< 0.001	< 0.001	< 0.001	< 0.001	< 0.001
**P-tau181**	Q2	-0.012 (-0.024 to 0.001)	-0.007 (-0.017 to 0.003)	-0.005 (-0.014 to 0.003)	-0.008 (-0.017 to 0.001)	-0.008 (-0.015 to -0.001)*
	Q3	-0.021 (-0.034 to -0.009)**	-0.020 (-0.030 to -0.010)***	-0.024 (-0.033 to -0.016)***	-0.025 (-0.034 to -0.016)***	-0.024 (-0.032 to -0.017)***
	Q4	-0.041 (-0.055 to -0.027)***	-0.039 (-0.050 to -0.028)***	-0.042 (-0.051 to -0.033)***	-0.037 (-0.047 to -0.028)***	-0.043 (-0.051 to -0.036)***
P trend		< 0.001	< 0.001	< 0.001	< 0.001	< 0.001
**T-tau**	Q2	-0.006 (-0.019 to 0.007)	-0.004 (-0.015 to 0.006)	-0.007 (-0.016 to 0.002)	-0.004 (-0.014 to 0.005)	-0.005 (-0.013 to 0.003)
	Q3	-0.011 (-0.024 to 0.002)	-0.011 (-0.021 to -0.000)*	-0.017 (-0.026 to -0.008)***	-0.004 (-0.013 to 0.006)	-0.012 (-0.020 to -0.005)**
	Q4	-0.011 (-0.025 to 0.002)	-0.016 (-0.026 to -0.005)**	-0.014 (-0.023 to -0.005)**	-0.011 (-0.021 to -0.002)*	-0.015 (-0.022 to -0.007)***
P trend		n.s.	< 0.01	< 0.001	<0.05	< 0.001
**NfL**	Q2	-0.022 (-0.034 to -0.009)**	-0.002 (-0.012 to 0.007)	-0.007 (-0.015 to 0.001)	-0.017 (-0.025 to -0.008)***	-0.011 (-0.018 to -0.004)**
	Q3	-0.020 (-0.032 to -0.007)**	-0.019 (-0.029 to -0.010)***	-0.023 (-0.032 to -0.015)***	-0.026 (-0.035 to -0.017)***	-0.022 (-0.029 to -0.015)***
	Q4	-0.050 (-0.065 to -0.035)***	-0.057 (-0.069 to -0.045)***	-0.060 (-0.069 to -0.050)***	-0.051 (-0.061 to -0.040)***	-0.057 (-0.065 to -0.049)***
P trend		< 0.001	< 0.001	< 0.001	< 0.001	< 0.001
**GFAP**	Q2	-0.002 (-0.015 to 0.010)	0.001 (-0.009 to 0.011)	-0.003 (-0.011 to 0.006)	-0.004 (-0.013 to 0.005)	-0.002 (-0.009 to 0.005)
	Q3	-0.012 (-0.025 to 0.000)	-0.016 (-0.025 to -0.006)**	-0.016 (-0.025 to -0.008)***	-0.014 (-0.023 to -0.005)**	-0.015 (-0.022 to -0.008)***
	Q4	-0.038 (-0.053 to -0.024)***	-0.042 (-0.054 to -0.031)***	-0.048 (-0.057 to -0.038)***	-0.037 (-0.048 to -0.028)***	-0.045 (-0.053 to -0.037)***
P trend		< 0.001	< 0.001	< 0.001	< 0.001	< 0.001

[a] For the Aβ42/40 ratio, quartiles are presented in reverse order relative to other the biomarkers (Q1 = lowest ratio/highest amyloid burden; Q4 = highest ratio/lowest amyloid burden [reference]) to facilitate comparisons across biomarkers. For all other biomarkers, Q1 is the reference.
* $P< 0.05$, ** $P< 0.01$, *** $P< 0.001$

© 2026 Laukka EJ et al. *JAMA Network Open*.

eTable 8. Estimates From Linear Mixed Models by Quartiles of 6 Individual Biomarkers of Alzheimer Disease for Global Cognition in Participants With Data on All 4 Cognitive Domains (n = 1935). Beta coefficients together with 95% confidence intervals represent differences in annual rate of decline compared to the reference quartile, indicating the lowest level of brain pathology. All models were controlled for age, sex, education, anemia, kidney disease, obesity, cerebrovascular disease, and cardiovascular disease.

		Global cognition
**Aβ42/Aβ40**	Q3[a]	-0.007 (-0.013 to -0.000)*
	Q2	-0.013 (-0.020 to -0.006)***
	Q1	-0.019 (-0.026 to -0.012)***
P trend		< 0.001
**P-tau217**	Q2	-0.009 (-0.015 to -0.003)**
	Q3	-0.020 (-0.026 to -0.013)***
	Q4	-0.044 (-0.052 to -0.036)***
P trend		< 0.001
**P-tau181**	Q2	-0.008 (-0.014 to -0.002)**
	Q3	-0.023 (-0.029 to -0.016)***
	Q4	-0.040 (-0.047 to -0.032)***
P trend		< 0.001
**T-tau**	Q2	-0.007 (-0.014 to -0.000)*
	Q3	-0.013 (-0.020 to -0.006)***
	Q4	-0.010 (-0.017 to -0.003)**
P trend		< 0.01
**NfL**	Q2	-0.012 (-0.018 to -0.006)***
	Q3	-0.021 (-0.028 to -0.015)***
	Q4	-0.051 (-0.059 to -0.043)***
P trend		< 0.001
**GFAP**	Q2	-0.004 (-0.010 to 0.002)
	Q3	-0.014 (-0.020 to -0.007)***
	Q4	-0.037 (-0.044 to -0.029)***
P trend		< 0.001

[a] For the Aβ42/Aβ40 ratio, quartiles are presented in reverse order relative to other the biomarkers (Q1 = lowest ratio/highest amyloid burden; Q4 = highest ratio/lowest amyloid burden [reference]) to facilitate comparisons across biomarkers. For all other biomarkers, Q1 is the reference.
* *P*< 0.05, ** *P*< 0.01, *** *P*< 0.001

© 2026 Laukka EJ et al. *JAMA Network Open*.

eTable 9. Estimates from Linear Mixed Models for 6 Continuous Individual Biomarkers of Alzheimer Disease in the Total Sample (N = 2008) for 4 Specific Cognitive Domains and Global Cognition. Beta coefficients together with 95% confidence intervals represent baseline differences and differences in annual rate of cognitive decline for each baseline biomarker unit change. All models were controlled for age, sex, education, anemia, kidney disease, obesity, cerebrovascular disease, and cardiovascular disease.

		**Episodic memory**	**Semantic memory**	**Verbal fluency**	**Perceptual speed**	**Global cognition**
**Aβ42/Aβ40**	Level	0.018 (-0.018 to 0.053)	-0.025 (-0.066 to 0.015)	-0.001 (-0.033 to 0.032)	-0.009 (-0.041 to 0.022)	-0.000 (-0.025 to 0.024)
	Change	0.008 (0.003 to 0.013)**	0.007 (0.003 to 0.011)***	0.006 (0.003 to 0.010)***	0.007 (0.003 to 0.011)***	0.008 (0.005 to 0.010)***
**P-tau217**	Level	-0.006 (-0.043 to 0.031)	0.005 (-0.037 to 0.047)	0.006 (-0.027 to 0.040)	0.005 (-0.028 to 0.038)	-0.004 (-0.029 to 0.021)
	Change	-0.037 (-0.047 to -0.028)***	-0.027 (-0.035 to -0.020)***	-0.034 (-0.041 to -0.028)***	-0.027 (-0.034 to -0.020)***	-0.036 (-0.042 to -0.031)***
**P-tau181**	Level	-0.012 (-0.058 to 0.035)	0.015 (-0.038 to 0.068)	0.017 (-0.025 to 0.059)	0.009 (-0.032 to 0.050)	-0.003 (-0.035 to 0.029)
	Change	-0.023 (-0.030 to -0.015)***	-0.021 (-0.027 to -0.015)***	-0.023 (-0.028 to -0.017)***	-0.020 (-0.025 to -0.014)***	-0.024 (-0.029 to -0.020)***
**T-tau**	Level	0.027 (-0.008 to 0.063)	0.029 (-0.012 to 0.069)	0.038 (0.005 to 0.070)*	0.010 (-0.022 to 0.041)	0.023 (-0.002 to 0.047)
	Change	-0.005 (-0.010 to 0.001)	-0.006 (-0.010 to -0.001)**	-0.005 (-0.009 to -0.001)**	-0.003 (-0.007 to 0.000)	-0.005 (-0.009 to -0.002)**
**NfL**	Level	0.010 (-0.036 to 0.057)	-0.037 (-0.089 to 0.015)	-0.013 (-0.054 to 0.028)	-0.040 (-0.082 to 0.001)	-0.039 (-0.070 to -0.008)*
	Change	-0.011 (-0.017 to -0.004)**	-0.016 (-0.022 to -0.011)***	-0.017 (-0.021 to -0.012)***	-0.012 (-0.017 to -0.008)***	-0.015 (-0.019 to -0.012)***
**GFAP**	Level	0.028 (-0.010 to 0.067)	0.056 (0.012 to 0.101)*	0.048 (0.012 to 0.083)**	0.000 (-0.034 to 0.035)	0.029 (0.002 to 0.056)*
	Change	-0.007 (-0.012 to -0.001)*	-0.006 (-0.010 to -0.002)**	-0.009 (-0.012 to -0.005)***	-0.006 (-0.010 to -0.002)**	-0.008 (-0.011 to -0.005)***

* $P < 0.05$, ** $P < 0.01$, *** $P < 0.001$

© 2026 Laukka EJ et al. *JAMA Network Open*.

eTable 10. Estimates from Linear Mixed Models for NfL and GFAP in the Total Sample (N = 2008) and in Individuals With Low (n = 1212) and High (n = 796) p-Tau217 for Global Cognition. Beta coefficients together with 95% confidence intervals represent baseline differences and differences in annual rate of cognitive decline for each baseline biomarker unit change. All models were controlled for age, sex, education, anemia, kidney disease, obesity, cerebrovascular disease, and cardiovascular disease.

		**Total sample**	**Low p-tau217**	**High p-tau217**
**NfL**	Level	-0.039 (-0.070 to -0.008)*	-0.015 (-0.076 to 0.045)	-0.053 (-0.092 to -0.015)**
	Change	-0.015 (-0.019 to -0.012)***	-0.020 (-0.025 to -0.014)***	-0.008 (-0.014 to -0.002)**
**GFAP**	Level	0.029 (0.002 to 0.056)*	0.024 (-0.031 to 0.080)	0.029 (-0.002 to 0.061)
	Change	-0.008 (-0.011 to -0.005)***	-0.015 (-0.021 to -0.010)***	-0.002 (-0.007 to 0.002)

* $P < 0.05$, ** $P < 0.01$, *** $P < 0.001$

© 2026 Laukka EJ et al. *JAMA Network Open*.

eTable 11. Estimates From Linear Mixed Models for 6 Continuous Individual Biomarkers of Alzheimer Disease in *APOE* ε4 Noncarriers (n = 1375) for 4 Specific Cognitive Domains and Global Cognition. Beta coefficients together with 95% confidence intervals represent baseline differences and differences in annual rate of cognitive decline for each baseline biomarker unit change. All models were controlled for age, sex, education, anemia, kidney disease, obesity, cerebrovascular disease, and cardiovascular disease.

		Episodic memory	Semantic memory	Verbal fluency	Perceptual speed	Global cognition
**Aβ42/Aβ40**	Level	0.010 (-0.037 to 0.058)	-0.008 (-0.064 to 0.048)	-0.013 (-0.058 to 0.031)	-0.012 (-0.055 to 0.031)	-0.001 (-0.034 to 0.032)
	Change	0.007 (0.002 to 0.013)**	0.007 (0.002 to 0.011)**	0.005 (0.001 to 0.009)**	0.006 (0.002 to 0.010)**	0.006 (0.003 to 0.010)***
**P-tau217**	Level	0.014 (-0.027 to 0.054)	0.003 (-0.043 to 0.049)	0.011 (-0.025 to 0.048)	0.010 (-0.025 to 0.046)	0.003 (-0.024 to 0.031)
	Change	-0.030 (-0.041 to -0.019)***	-0.024 (-0.033 to -0.015)***	-0.030 (-0.038 to -0.022)***	-0.024 (-0.033 to -0.016)***	-0.032 (-0.039 to -0.026)***
**P-tau181**	Level	0.000 (-0.052 to 0.052)	0.023 (-0.038 to 0.083)	0.035 (-0.013 to 0.083)	0.025 (-0.021 to 0.072)	0.011 (-0.024 to 0.047)
	Change	-0.018 (-0.027 to -0.010)***	-0.018 (-0.025 to -0.011)***	-0.019 (-0.025 to -0.013)***	-0.017 (-0.024 to -0.011)***	-0.020 (-0.025 to -0.015)***
**T-tau**	Level	0.030 (-0.012 to 0.072)	0.009 (-0.039 to 0.058)	0.026 (-0.013 to 0.064)	0.004 (-0.034 to 0.041)	0.014 (-0.015 to 0.043)
	Change	-0.006 (-0.013 to 0.000)	-0.007 (-0.013 to -0.002)**	-0.007 (-0.012 to -0.002)**	-0.006 (-0.011 to -0.001)*	-0.008 (-0.012 to -0.004)***
**NfL**	Level	0.026 (-0.033 to 0.085)	-0.034 (-0.101 to 0.033)	-0.046 (-0.098 to 0.006)	-0.042 (-0.095 to 0.011)	-0.047 (-0.086 to -0.007)*
	Change	-0.019 (-0.028 to -0.010)***	-0.029 (-0.036 to -0.021)***	-0.027 (-0.033 to -0.021)***	-0.025 (-0.031 to -0.018)***	-0.025 (-0.031 to -0.020)***
**GFAP**	Level	0.038 (-0.012 to 0.089)	0.072 (0.013 to 0.130)*	0.026 (-0.021 to 0.072)	-0.003 (-0.048 to 0.042)	0.027 (-0.008 to 0.062)
	Change	-0.015 (-0.024 to -0.006)**	-0.013 (-0.020 to -0.005)**	-0.018 (-0.024 to -0.012)***	-0.018 (-0.024 to -0.011)***	-0.017 (-0.022 to -0.012)***

* $P< 0.05$, ** $P< 0.01$, *** $P< 0.001$

© 2026 Laukka EJ et al. *JAMA Network Open*.

eTable 12. Estimates From Linear Mixed Models for 6 Continuous Individual Biomarkers of Alzheimer Disease in *APOE* ε4 Carriers (n = 579) for 4 Specific Cognitive Domains and Global Cognition. Beta coefficients together with 95% confidence intervals represent baseline differences and differences in annual rate of cognitive decline for each baseline biomarker unit change. All models were controlled for age, sex, education, anemia, kidney disease, obesity, cerebrovascular disease, and cardiovascular disease.

		Episodic memory	Semantic memory	Verbal fluency	Perceptual speed	Global cognition
**Aβ42/Aβ40**	Level	0.014 (-0.040 to 0.068)	-0.058 (-0.116 to -0.001)*	0.000 (-0.048 to 0.049)	-0.024 (-0.071 to 0.023)	-0.013 (-0.049 to 0.023)
	Change	0.009 (-0.002 to 0.021)	0.008 (-0.001 to 0.017)	0.011 (0.003 to 0.018)**	0.011 (0.003 to 0.019)**	0.011 (0.004 to 0.018)**
**P-tau217**	Level	-0.109 (-0.216 to -0.003)*	0.012 (-0.102 to 0.126)	-0.016 (-0.110 to 0.078)	0.010 (-0.082 to 0.102)	-0.040 (-0.111 to 0.031)
	Change	-0.047 (-0.068 to -0.026)***	-0.038 (-0.055 to -0.021)***	-0.043 (-0.057 to -0.030)***	-0.032 (-0.046 to -0.017)***	-0.043 (-0.054 to -0.031)***
**P-tau181**	Level	-0.065 (-0.180 to 0.050)	-0.036 (-0.157 to 0.085)	-0.052 (-0.152 to 0.048)	-0.050 (-0.148 to 0.049)	-0.066 (-0.142 to 0.009)
	Change	-0.036 (-0.054 to -0.017)***	-0.035 (-0.049 to -0.020)***	-0.035 (-0.047 to -0.023)***	-0.028 (-0.040 to -0.015)***	-0.037 (-0.047 to -0.026)***
**T-tau**	Level	0.022 (-0.048 to 0.093)	0.080 (0.005 to 0.155)*	0.076 (0.013 to 0.139)*	0.031 (-0.030 to 0.092)	0.049 (0.002 to 0.096)*
	Change	-0.002 (-0.011 to 0.007)	-0.004 (-0.011 to 0.003)	-0.003 (-0.009 to 0.003)	-0.000 (-0.007 to 0.006)	-0.002 (-0.008 to 0.003)
**NfL**	Level	-0.014 (-0.091 to 0.062)	-0.067 (-0.148 to 0.014)	0.024 (-0.045 to 0.092)	-0.043 (-0.109 to 0.023)	-0.036 (-0.087 to 0.014)
	Change	-0.002 (-0.012 to 0.008)	-0.006 (-0.013 to 0.002)	-0.006 (-0.013 to 0.000)	-0.002 (-0.008 to 0.005)	-0.005 (-0.010 to 0.001)
**GFAP**	Level	0.022 (-0.039 to 0.084)	0.044 (-0.022 to 0.110)	0.086 (0.031 to 0.141)**	0.015 (-0.039 to 0.068)	0.040 (-0.001 to 0.081)
	Change	-0.002 (-0.009 to 0.004)	-0.003 (-0.009 to 0.002)	-0.005 (-0.010 to 0.000)	-0.001 (-0.006 to 0.004)	-0.003 (-0.007 to 0.001)

* $P < 0.05$, ** $P < 0.01$, *** $P < 0.001$

© 2026 Laukka EJ et al. *JAMA Network Open*.

eTable 13. Estimates From Linear Mixed Models for 6 Continuous Individual Biomarkers of Alzheimer Disease in Males (n = 793) for 4 Specific Cognitive Domains and Global Cognition. Beta coefficients together with 95% confidence intervals represent baseline differences and differences in annual rate of cognitive decline for each baseline biomarker unit change. All models were controlled for age, sex, education, anemia, kidney disease, obesity, cerebrovascular disease, and cardiovascular disease.

		Episodic memory	Semantic memory	Verbal fluency	Perceptual speed	Global cognition
**Aβ42/Aβ40**	Level	0.040 (-0.024 to 0.104)	-0.011 (-0.086 to 0.064)	-0.013 (-0.072 to 0.046)	-0.006 (-0.067 to 0.056)	0.006 (-0.039 to 0.051)
	Change	0.005 (-0.003 to 0.013)	0.009 (0.003 to 0.015)**	0.008 (0.002 to 0.014)**	0.005 (-0.001 to 0.012)	0.007 (0.003 to 0.012)**
**P-tau217**	Level	0.019 (-0.023 to 0.061)	0.018 (-0.030 to 0.066)	0.005 (-0.033 to 0.042)	-0.002 (-0.041 to 0.036)	0.007 (-0.021 to 0.036)
	Change	-0.032 (-0.047 to -0.017)***	-0.028 (-0.040 to -0.015)***	-0.034 (-0.044 to -0.023)***	-0.029 (-0.041 to -0.017)***	-0.033 (-0.042 to -0.024)***
**P-tau181**	Level	0.021 (-0.039 to 0.081)	0.049 (-0.020 to 0.118)	-0.003 (-0.057 to 0.051)	0.016 (-0.040 to 0.072)	0.016 (-0.025 to 0.057)
	Change	-0.019 (-0.031 to -0.008)**	-0.019 (-0.028 to -0.010)***	-0.019 (-0.027 to -0.011)***	-0.022 (-0.031 to -0.013)***	-0.021 (-0.028 to -0.014)***
**T-tau**	Level	0.016 (-0.027 to 0.059)	0.023 (-0.029 to 0.075)	0.017 (-0.023 to 0.058)	0.008 (-0.034 to 0.050)	0.022 (-0.009 to 0.053)
	Change	-0.000 (-0.008 to 0.007)	-0.002 (-0.008 to 0.003)	-0.000 (-0.005 to 0.005)	-0.001 (-0.007 to 0.004)	-0.003 (-0.007 to 0.002)
**Nfl**	Level	0.044 (-0.020 to 0.107)	-0.019 (-0.089 to 0.051)	0.014 (-0.041 to 0.068)	-0.042 (-0.102 to 0.017)	-0.019 (-0.060 to 0.023)
	Change	-0.008 (-0.017 to -0.000)*	-0.009 (-0.016 to -0.003)**	-0.010 (-0.015 to -0.004)**	-0.006 (-0.012 to 0.000)	-0.009 (-0.014 to -0.004)***
**GFAP**	Level	0.042 (-0.001 to 0.085)	0.064 (0.014 to 0.114)*	0.052 (0.013 to 0.092)**	0.009 (-0.031 to 0.050)	0.041 (0.011 to 0.071)**
	Change	-0.005 (-0.010 to 0.001)	-0.003 (-0.007 to 0.002)	-0.004 (-0.008 to 0.000)	-0.002 (-0.006 to 0.003)	-0.004 (-0.007 to -0.000)*

* $P< 0.05$, ** $P< 0.01$, *** $P< 0.001$

© 2026 Laukka EJ et al. *JAMA Network Open*.

eTable 14. Estimates From Linear Mixed Models for 6 Continuous Individual Biomarkers of Alzheimer Disease in Females (n = 1215) for 4 Specific Cognitive Domains and Global Cognition. Beta coefficients together with 95% confidence intervals represent baseline differences and differences in annual rate of cognitive decline for each baseline biomarker unit change. All models were controlled for age, sex, education, anemia, kidney disease, obesity, cerebrovascular disease, and cardiovascular disease.

		**Episodic memory**	**Semantic memory**	**Verbal fluency**	**Perceptual speed**	**Global cognition**
**Aβ42/Aβ40**	Level	0.009 (-0.033 to 0.052)	-0.028 (-0.076 to 0.020)	0.005 (-0.033 to 0.044)	-0.013 (-0.049 to 0.023)	-0.001 (-0.030 to 0.028)
	Change	0.009 (0.003 to 0.015)**	0.006 (0.001 to 0.011)*	0.006 (0.001 to 0.010)*	0.008 (0.003 to 0.012)**	0.008 (0.004 to 0.011)***
**P-tau217**	Level	-0.108 (-0.191 to -0.026)*	-0.090 (-0.184 to 0.005)	-0.007 (-0.083 to 0.069)	0.031 (-0.042 to 0.104)	-0.069 (-0.125 to -0.012)*
	Change	-0.037 (-0.050 to -0.024)***	-0.028 (-0.038 to -0.017)***	-0.035 (-0.044 to -0.026)***	-0.026 (-0.035 to -0.017)***	-0.038 (-0.046 to -0.031)***
**P-tau181**	Level	-0.072 (-0.146 to 0.003)	-0.068 (-0.153 to 0.016)	0.034 (-0.034 to 0.103)	-0.013 (-0.077 to 0.051)	-0.049 (-0.100 to 0.003)
	Change	-0.023 (-0.034 to -0.012)***	-0.022 (-0.031 to -0.014)***	-0.026 (-0.033 to -0.019)***	-0.017 (-0.025 to -0.010)***	-0.026 (-0.032 to -0.020)***
**T-tau**	Level	-0.011 (-0.069 to 0.048)	0.017 (-0.048 to 0.083)	0.062 (0.009 to 0.114)*	0.011 (-0.039 to 0.062)	0.014 (-0.025 to 0.054)
	Change	-0.004 (-0.012 to 0.004)	-0.009 (-0.015 to -0.003)**	-0.010 (-0.016 to -0.005)***	-0.006 (-0.012 to -0.000)*	-0.009 (-0.013 to -0.004)***
**Nfl**	Level	-0.026 (-0.093 to 0.042)	-0.059 (-0.136 to 0.019)	-0.051 (-0.113 to 0.011)	-0.037 (-0.096 to 0.021)	-0.065 (-0.111 to -0.018)**
	Change	-0.015 (-0.025 to -0.005)**	-0.027 (-0.035 to -0.019)***	-0.026 (-0.032 to -0.019)***	-0.021 (-0.028 to -0.014)***	-0.023 (-0.029 to -0.018)***
**GFAP**	Level	-0.023 (-0.109 to 0.063)	0.022 (-0.075 to 0.119)	0.015 (-0.064 to 0.093)	-0.037 (-0.110 to 0.036)	-0.021 (-0.080 to 0.038)
	Change	-0.014 (-0.025 to -0.002)*	-0.018 (-0.027 to -0.009)***	-0.025 (-0.032 to -0.017)***	-0.021 (-0.029 to -0.013)***	-0.021 (-0.028 to -0.015)***

* $P < 0.05$, ** $P < 0.01$, *** $P < 0.001$

© 2026 Laukka EJ et al. *JAMA Network Open*.

eTable 15. Estimates From Linear Mixed Models for 6 Continuous Individual Biomarkers of Alzheimer Disease in Young-Old (Aged <78 Years, n = 1294) for 4 Specific Cognitive Domains and Global Cognition. Beta coefficients together with 95% confidence intervals represent baseline differences and differences in annual rate of cognitive decline for each baseline biomarker unit change. All models were controlled for age, sex, education, anemia, kidney disease, obesity, cerebrovascular disease, and cardiovascular disease.

		**Episodic memory**	**Semantic memory**	**Verbal fluency**	**Perceptual speed**	**Global cognition**
**Aβ42/Aβ40**	Level	0.021 (-0.021 to 0.063)	-0.026 (-0.074 to 0.023)	0.002 (-0.036 to 0.040)	-0.019 (-0.060 to 0.021)	-0.002 (-0.031 to 0.026)
	Change	0.008 (0.002 to 0.014)**	0.005 (0.001 to 0.009)*	0.006 (0.002 to 0.009)**	0.006 (0.002 to 0.010)**	0.007 (0.004 to 0.010)***
**P-tau217**	Level	-0.029 (-0.141 to 0.083)	0.113 (-0.017 to 0.243)	0.089 (-0.014 to 0.192)	0.049 (-0.061 to 0.159)	0.045 (-0.032 to 0.123)
	Change	-0.031 (-0.044 to -0.017)***	-0.018 (-0.028 to -0.008)***	-0.025 (-0.034 to -0.016)***	-0.020 (-0.031 to -0.009)***	-0.025 (-0.032 to -0.017)***
**P-tau181**	Level	-0.005 (-0.094 to 0.083)	0.058 (-0.045 to 0.162)	0.080 (-0.002 to 0.161)	0.026 (-0.060 to 0.113)	0.030 (-0.032 to 0.091)
	Change	-0.016 (-0.026 to -0.005)**	-0.015 (-0.022 to -0.007)***	-0.017 (-0.023 to -0.010)***	-0.015 (-0.023 to -0.008)***	-0.016 (-0.021 to -0.011)***
**T-tau**	Level	0.045 (-0.009 to 0.099)	0.089 (0.027 to 0.152)**	0.071 (0.022 to 0.120)**	0.040 (-0.013 to 0.093)	0.061 (0.024 to 0.099)**
	Change	-0.005 (-0.011 to 0.002)	-0.005 (-0.009 to -0.000)*	-0.004 (-0.008 to 0.000)	-0.002 (-0.007 to 0.003)	-0.004 (-0.007 to -0.001)*
**NfL**	Level	0.009 (-0.065 to 0.083)	0.000 (-0.086 to 0.086)	0.035 (-0.032 to 0.103)	-0.059 (-0.130 to 0.013)	-0.012 (-0.063 to 0.039)
	Change	-0.007 (-0.016 to 0.003)	-0.006 (-0.012 to 0.001)	-0.010 (-0.016 to -0.004)***	-0.007 (-0.014 to -0.001)*	-0.007 (-0.012 to -0.003)**
**GFAP**	Level	0.003 (-0.054 to 0.060)	0.046 (-0.020 to 0.112)	0.061 (0.009 to 0.113)*	-0.022 (-0.077 to 0.033)	0.021 (-0.018 to 0.061)
	Change	-0.002 (-0.008 to 0.004)	-0.003 (-0.008 to 0.001)	-0.005 (-0.009 to -0.001)**	-0.003 (-0.007 to 0.002)	-0.004 (-0.007 to -0.000)*

* $P < 0.05$, ** $P < 0.01$, *** $P < 0.001$

© 2026 Laukka EJ et al. *JAMA Network Open*.

eTable 16. Estimates From Linear Mixed Models for 6 Continuous Individual Biomarkers of Alzheimer Disease in Old-Old (Aged ≥78 Years, n = 714) for 4 Specific Cognitive Domains and Global Cognition. Beta coefficients together with 95% confidence intervals represent baseline differences and differences in annual rate of cognitive decline for each baseline biomarker unit change. All models were controlled for age, sex, education, anemia, kidney disease, obesity, cerebrovascular disease, and cardiovascular disease.

		Episodic memory	Semantic memory	Verbal fluency	Perceptual speed	Global cognition
**Aβ42/Aβ40**	Level	0.026 (-0.053 to 0.104)	-0.012 (-0.098 to 0.074)	0.021 (-0.053 to 0.095)	0.037 (-0.047 to 0.121)	0.018 (-0.039 to 0.075)
	Change	0.004 (-0.009 to 0.017)	0.013 (0.001 to 0.026)*	0.006 (-0.004 to 0.016)	0.012 (0.001 to 0.024)*	0.008 (-0.000 to 0.017)
**P-tau217**	Level	-0.006 (-0.046 to 0.035)	-0.007 (-0.051 to 0.038)	-0.015 (-0.053 to 0.023)	-0.006 (-0.049 to 0.036)	-0.011 (-0.040 to 0.019)
	Change	-0.039 (-0.058 to -0.020)***	-0.028 (-0.047 to -0.008)**	-0.031 (-0.046 to -0.016)***	-0.030 (-0.048 to -0.012)**	-0.039 (-0.052 to -0.026)***
**P-tau181**	Level	-0.025 (-0.082 to 0.033)	-0.010 (-0.072 to 0.053)	-0.034 (-0.088 to 0.019)	-0.011 (-0.071 to 0.049)	-0.022 (-0.063 to 0.019)
	Change	-0.033 (-0.051 to -0.016)***	-0.022 (-0.039 to -0.005)*	-0.020 (-0.033 to -0.006)**	-0.026 (-0.042 to -0.010)**	-0.029 (-0.040 to -0.017)***
**T-tau**	Level	0.005 (-0.045 to 0.056)	-0.022 (-0.077 to 0.032)	-0.004 (-0.050 to 0.043)	-0.021 (-0.073 to 0.031)	-0.012 (-0.048 to 0.023)
	Change	0.001 (-0.013 to 0.015)	-0.005 (-0.019 to 0.009)	-0.005 (-0.016 to 0.006)	-0.007 (-0.021 to 0.006)	-0.005 (-0.016 to 0.005)
**NfL**	Level	-0.005 (-0.070 to 0.060)	-0.060 (-0.128 to 0.008)	-0.091 (-0.148 to -0.033)**	-0.063 (-0.131 to 0.005)	-0.072 (-0.117 to -0.027)**
	Change	-0.006 (-0.019 to 0.007)	-0.020 (-0.032 to -0.008)**	-0.011 (-0.021 to -0.001)*	-0.011 (-0.023 to 0.000)	-0.014 (-0.023 to -0.006)**
**GFAP**	Level	0.047 (-0.010 to 0.104)	0.050 (-0.012 to 0.112)	0.016 (-0.037 to 0.070)	0.011 (-0.048 to 0.070)	0.030 (-0.011 to 0.071)
	Change	-0.016 (-0.031 to -0.001)*	-0.002 (-0.017 to 0.013)	-0.008 (-0.020 to 0.004)	-0.010 (-0.024 to 0.003)	-0.011 (-0.021 to -0.000)*

* $P < 0.05$, ** $P < 0.01$, *** $P < 0.001$

© 2026 Laukka EJ et al. *JAMA Network Open*.

eFigure 1. Flow Chart of Study Participation

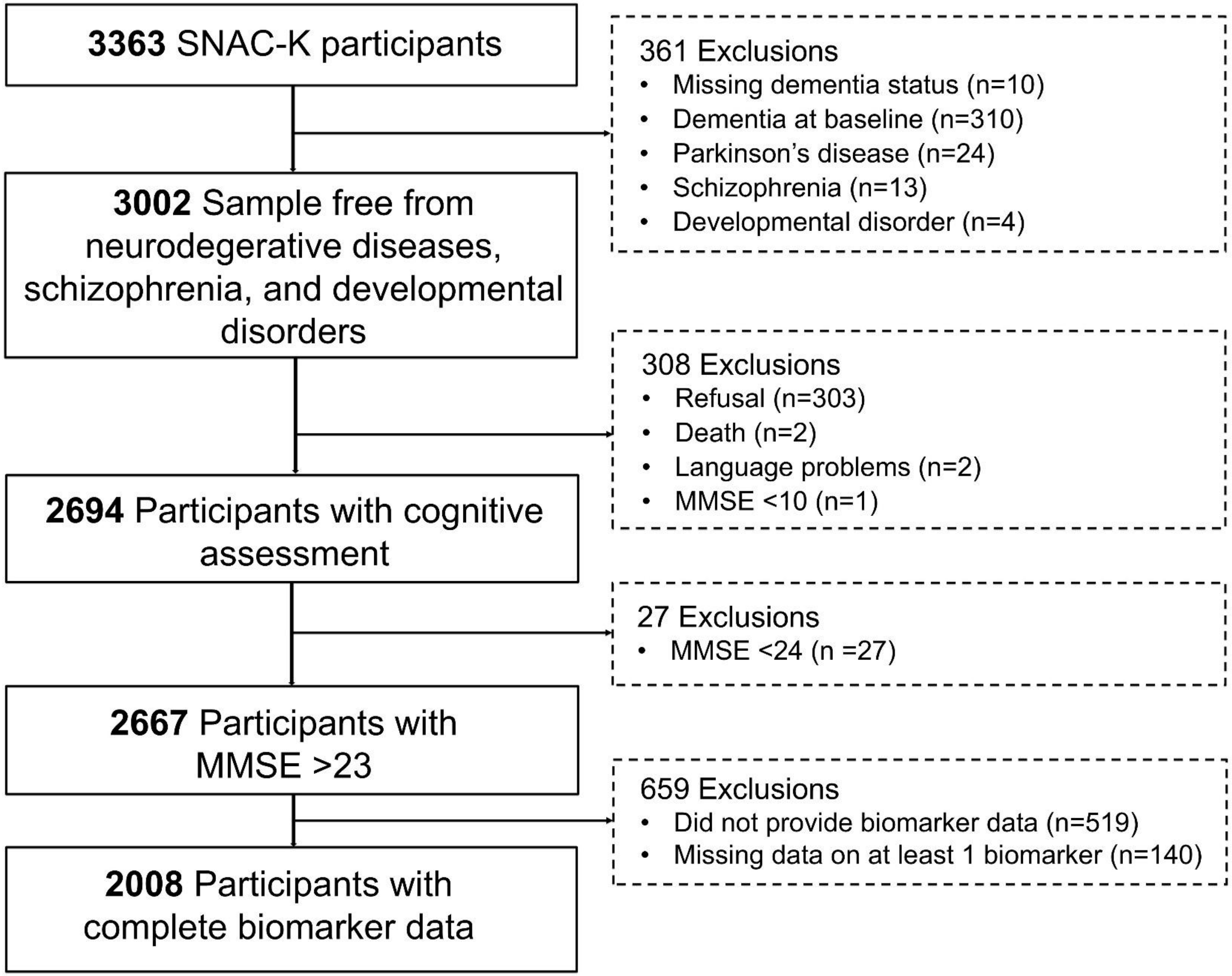

SNAC-K, Swedish National Study on Aging and Care-Kungsholmen; MMSE, Mini-Mental State Examination.

© 2026 Laukka EJ et al. *JAMA Network Open*.

eFigure 2. Estimated Trajectories of Episodic Memory, Semantic Memory, Verbal Fluency, and Perceptual Speed Across 15 Years for Quartiles of 6 Blood-Based Biomarkers of Alzheimer Disease

Panel A. Episodic memory

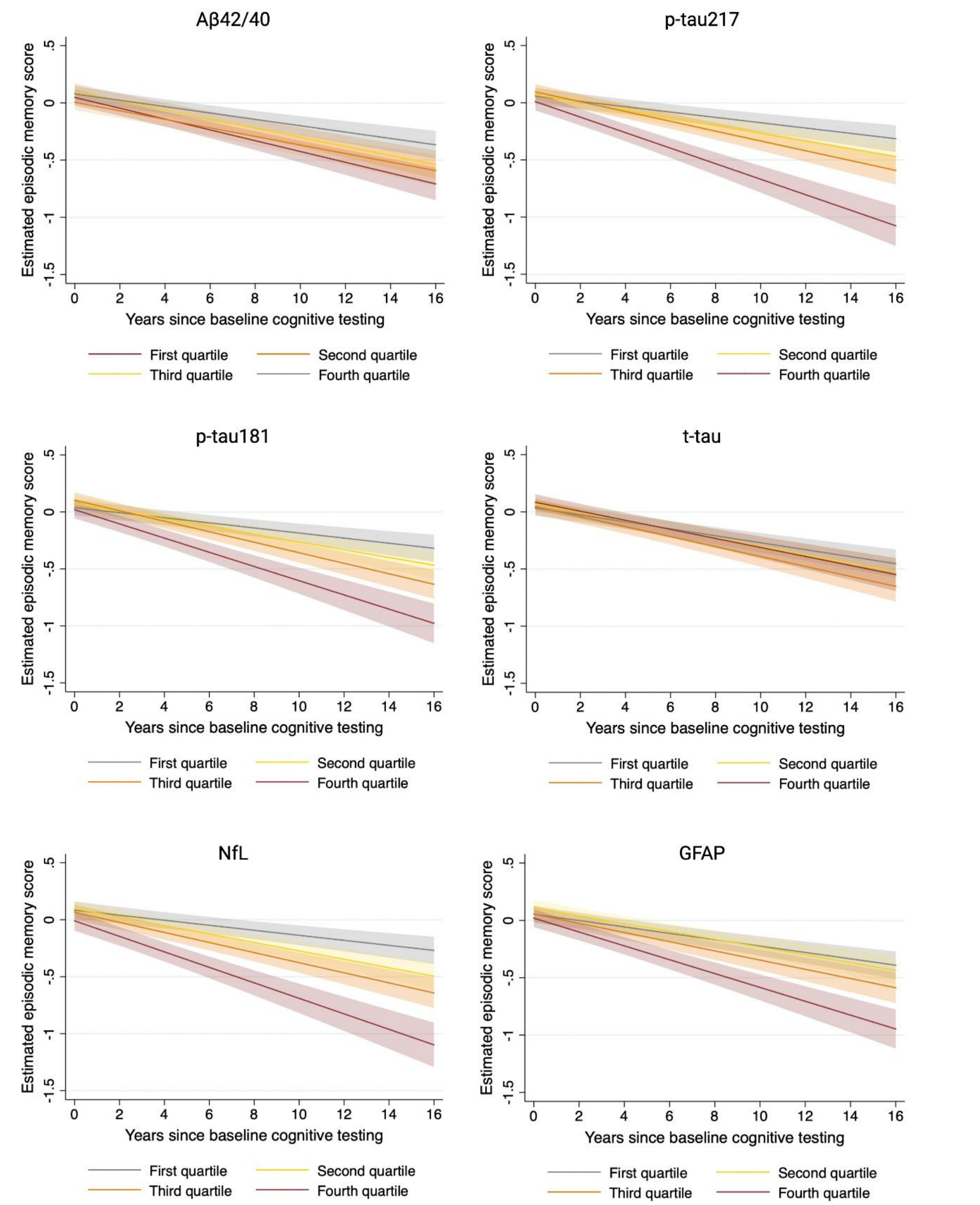

© 2026 Laukka EJ et al. *JAMA Network Open*.

Panel B. Semantic memory

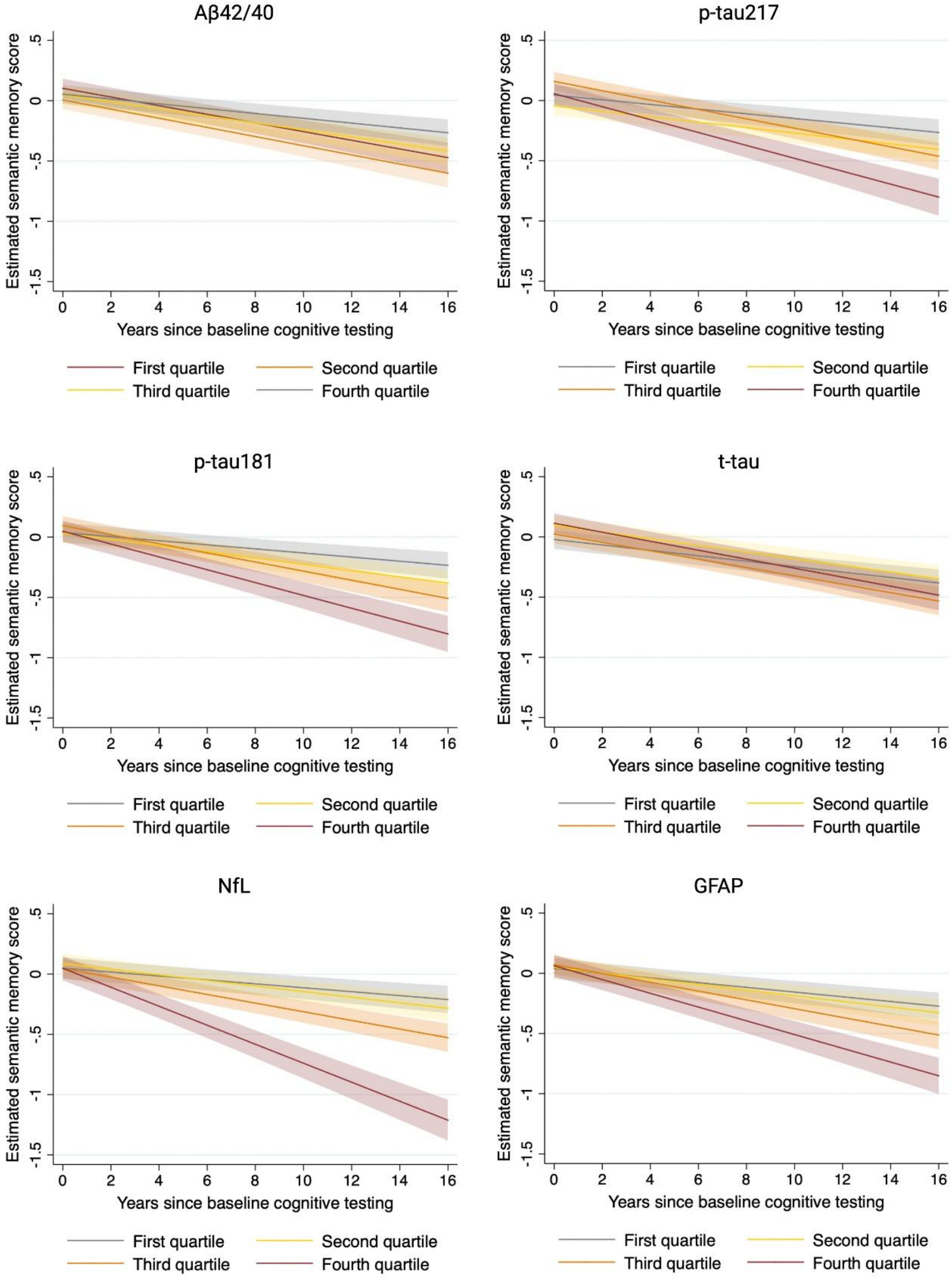

© 2026 Laukka EJ et al. *JAMA Network Open*.

Panel C. Verbal fluency

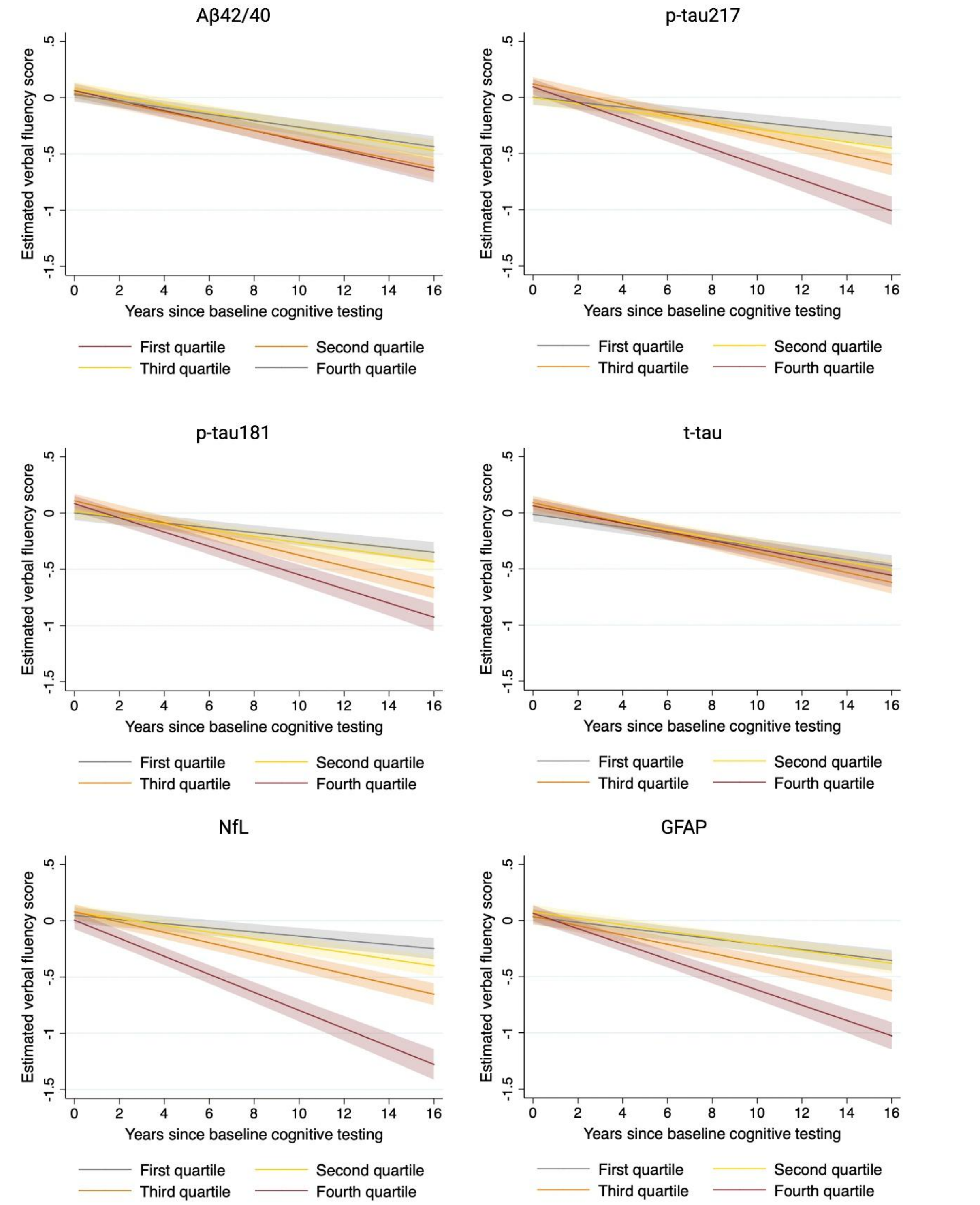

© 2026 Laukka EJ et al. *JAMA Network Open*.

Panel D. Perceptual speed

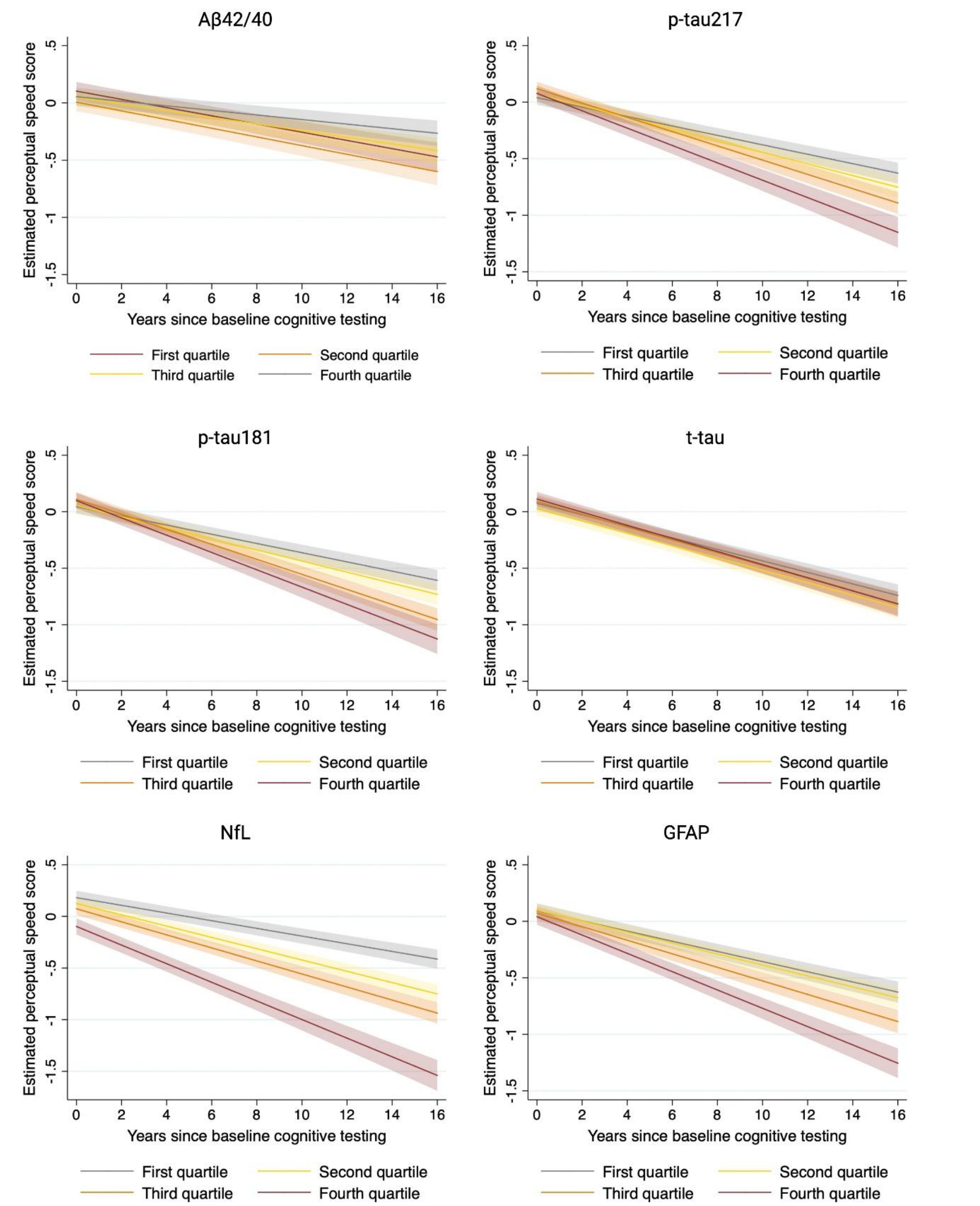

Note. All models were adjusted for age, sex education, anemia, kidney disease, obesity, cerebrovascular disease, and cardiovascular disease. For the Aβ42/40 ratio, Q4 = highest ratio/lowest amyloid burden is the reference. For all other biomarkers, Q1 is the reference.

© 2026 Laukka EJ et al. *JAMA Network Open*.

eMethods 1. Dementia Diagnosis

Dementia diagnosis in SNAC-K follows a rigorous three-step procedure. The physician conducts a face-to-face clinical assessment, including a neurological examination and assessment of clinical history, drug use, and cognitive functioning. Functional status, including both basic and instrumental activities of daily living (ADL and IADL), is part of the diagnostic process and when needed, proxy interviews are conducted to complement the clinical information. In step 1, the examining physician provides a preliminary diagnosis. Second, an independent physician, blinded to the initial evaluation, reviews the data documented from the examination and provides a second preliminary diagnosis. In case of disagreement, a senior neurologist with expertise in cognitive disorders, external to the data collection, establishes the final diagnosis.

eMethods 2. Classification of Diseases

Chronic diseases were ascertained based on a combination of data collected during the SNAC-K clinical examination, blood sampling, and proxy interviews, together with data from medical records and patient registries. It included participant and proxy self-reports, medical history, medication use, medical examination, and review of medical charts, and were further complemented through linkage with the Swedish National Patient Register. This has been detailed in a previous study (ref: Calderón Larrañaga A, Vetrano DL, Onder G, et al. Assessing and measuring chronic multimorbidity in the older population: A proposal for its operationalization. *J Gerontol A Biol Sci Med Sci.* 2017;72:1417-1423). For selected conditions, clinical and laboratory parameters were used to support the diagnosis: anemia was defined as hemoglobin <13 g/dL in men and <12 g/dL in women; chronic kidney disease as an estimated glomerular filtration rate <60 mL/min/1.73 m² (calculated using the 2009 CKD-EPI equation); and obesity as a body mass index ≥30 kg/m². Cerebrovascular diseases included transient ischemic attacks, stroke (ischemic and hemorrhagic), and intracranial hemorrhages, while cardiovascular diseases included atrial fibrillation, heart failure, and ischemic heart disease. All diagnoses were coded according to the International Classification of Diseases, Tenth Revision (ICD-10).

eMethods 3. Details on Protein Quantification

Serum Aβ40, Aβ42, and t-tau were measured using Simoa Neuro 3-plex A Kit (Quanterix, cat. no. 101995). Serum p-tau217 was quantified using the commercial assay Simoa® ALZpath p-tau217 Advantage PLUS (Quanterix, cat. no. 104570), and serum p-tau181 using Simoa p-tau181 Advantage V2 Kit (Quanterix, cat. no. 103714). Serum NfL and GFAP were measured using Simoa Neuro 2-plex B Kit (Quanterix, cat. no. 103520). For each kit, 25 µl of sample was diluted 1:4, and assays were performed according to the manufacturer's instructions or internal validation protocols. The Quanterix instrument calculates average enzyme per bead values for calibrators, controls, and samples across all proteins. The Quanterix SR-X software automatically performs curve-fitting and concentration extrapolation, generating graphical representations using the calibrators and a four-parameter logistic curve fit.

The median interval between the blood collection and cognitive assessment was 0.05 years (range -0.04 to 1.62 years).

© 2026 Laukka EJ et al. *JAMA Network Open*.
