# Supplementary material for: Blood Biomarkers of Alzheimer Disease and Rates of Global and Domain-Specific Cognitive Decline
Source: JAMA Netw Open. 2026 Aug 13;9(8):e2628975. doi: 10.1001/jamanetworkopen.2026.28975 (PMC13474032; doi:10.1001/jamanetworkopen.2026.28975)
Supplement: Supplementary file 2 [file jamanetwopen-e2628975-s002.pdf]

Data Sharing Statement

Data
Data available: No

Additional Information
Explanation for why data not available: Data related to the current study were derived from SNAC-K. Access to these anonymized data are available upon reasonable request and approval by the SNAC-K data management and maintenance committee (https://www.snac-k.se/for-researchers/application-form/).
